# Smartphone-Based Monitoring of Objective and Subjective Data in Affective Disorders: Where Are We and Where Are We Going? Systematic Review

**DOI:** 10.2196/jmir.7006

**Published:** 2017-07-24

**Authors:** Ezgi Dogan, Christian Sander, Xenija Wagner, Ulrich Hegerl, Elisabeth Kohls

**Affiliations:** ^1^ Medical Faculty Department of Psychiatry and Psychotherapy University Leipzig Leipzig Germany; ^2^ Depression Research Centre German Depression Foundation Leipzig Germany

**Keywords:** review, mood disorders, smartphone, ecological momentary assessment

## Abstract

**Background:**

Electronic mental health interventions for mood disorders have increased rapidly over the past decade, most recently in the form of various systems and apps that are delivered via smartphones.

**Objective:**

We aim to provide an overview of studies on smartphone-based systems that combine subjective ratings with objectively measured data for longitudinal monitoring of patients with affective disorders. Specifically, we aim to examine current knowledge on: (1) the feasibility of, and adherence to, such systems; (2) the association of monitored data with mood status; and (3) the effects of monitoring on clinical outcomes.

**Methods:**

We systematically searched PubMed, Web of Science, PsycINFO, and the Cochrane Central Register of Controlled Trials for relevant articles published in the last ten years (2007-2017) by applying Boolean search operators with an iterative combination of search terms, which was conducted in February 2017. Additional articles were identified via pearling, author correspondence, selected reference lists, and trial protocols.

**Results:**

A total of 3463 unique records were identified. Twenty-nine studies met the inclusion criteria and were included in the review. The majority of articles represented feasibility studies (n=27); two articles reported results from one randomized controlled trial (RCT). In total, six different self-monitoring systems for affective disorders that used subjective mood ratings and objective measurements were included. These objective parameters included physiological data (heart rate variability), behavioral data (phone usage, physical activity, voice features), and context/environmental information (light exposure and location). The included articles contained results regarding feasibility of such systems in affective disorders, showed reasonable accuracy in predicting mood status and mood fluctuations based on the objectively monitored data, and reported observations about the impact of monitoring on clinical state and adherence of patients to the system usage.

**Conclusions:**

The included observational studies and RCT substantiate the value of smartphone-based approaches for gathering long-term objective data (aside from self-ratings to monitor clinical symptoms) to predict changes in clinical states, and to investigate causal inferences about state changes in patients with affective disorders. Although promising, a much larger evidence-base is necessary to fully assess the potential and the risks of these approaches. Methodological limitations of the available studies (eg, small sample sizes, variations in the number of observations or monitoring duration, lack of RCT, and heterogeneity of methods) restrict the interpretability of the results. However, a number of study protocols stated ambitions to expand and intensify research in this emerging and promising field.

## Introduction

Unipolar depressive disorder is one of the leading chronic medical conditions in Europe, accounting for 13.7% of the disability burden [1], and is projected by the World Health Organization to become the second highest condition leading to burden of disease by 2030 [2]. International lifetime prevalence rates of depression range from 6.3-10.3% [3], and comorbidity [3] and mortality [4,5] are common; these disorders also incur a considerable economic burden [6]. Unipolar depression often takes a chronic recurrent course, with more than 70% of patients suffering several episodes throughout their lifetimes [7,8]. Bipolar disorder affects 2-2.5% of the adult population [9,10] and is considered a chronic disorder [11]. Both unipolar and bipolar affective disorders have severe consequences on functioning and quality of life [12]. Although effective treatment is available [13], it is estimated that at least 50% of patients with depression receive no treatment at all [14,15]. Furthermore, most patients do not adhere to medication regimens and referrals [16,17]. Barriers for help-seeking and treatment adherence include stigmatization, fear of discrimination, and an unavailability of services [18,19].

Electronic mental health (e-mental health) interventions represent a promising means of overcoming such barriers and increasing the capacity for patients’ self-management of depression [20]. Using the Internet to deliver treatment for affective disorders has been shown to be an effective option for reaching patients who were not able to receive face-to-face treatment due to geographical or other situational barriers [21], or to augment face-to-face therapy [22]. Internet availability in developed countries has expanded rapidly and now reaches individuals in nearly all social, age, ethnic, and education-level groups [23]. It is of promise for e-mental health interventions that people suffering from mental disorders actively use the Internet just as often as those in the general population who do not have mental illnesses (80%) [24]. A newer technological development is the rapid spread and technological improvement of smartphones. In the first quarter of 2016, there were 7.4 billion mobile phone subscriptions worldwide, of which 3.4 billion were for smartphones [25]. These numbers are estimated to reach 9 billion mobile phone and 6.3 billion smartphone subscriptions by the year 2021 [25]. Over 80% of the general population in Western countries already use mobile phones [23], thereby passively collecting an extensive amount of data that is not yet sufficiently used for medical and scientific purposes.

Capitalizing on these technological advances, a novel approach in e-mental health has emerged with the development and testing of several technology-enhanced systems for affective disorders that rely on combinations of subjective (ie, self-reported) and objective data, measured by sensors embedded within (or connected to) smartphones. When considering subjective self-ratings, smartphone apps result in better adherence and less missing data compared with paper-pencil or personal computer-based questionnaires or diaries [26]. However, the strongest asset of smartphone-based systems may be their high suitability for collecting objective data from behavioral and physiological monitoring. Differences in subjective (ie, psychological) and objective (ie, behavioral or physiological) data are a common finding (eg, pronounced differences in self-ratings on sleep quality compared to objectively recorded sleep quality are often seen in subjects with sleep disorders, such as insomniac patients who typically overestimate their actual wake times during the night). This issue has sparked a discussion about whether or not both approaches are alternatives or complement each other by capturing different aspects of the respective construct (a detailed discussion of this topic can be found in Tams et al [27]). Using both self-reported and objectively measured data derived from the smartphone-based systems may help to better monitor disease progression and optimize self-management approaches. Modern smartphones are able to incorporate a large number of various sensors (eg, global positioning system [GPS], Bluetooth, accelerometers, microphones), are carried mostly close to the body, have long lasting batteries, and contain large memory storage and powerful central processing units for recording and processing information [28,29]. Mobile technology has the vast advantage that it can be easily integrated into patients’ lives as a comfortable, simple, time-unconstrained, user-friendly, economical, and noninvasive method of registering and monitoring relevant signs and symptoms. Such technologies can be used to provide continuous self-management and psycho-educational content tailored to the specific needs of each individual on the basis of data registered on their own smartphones [23].

Using objective data for symptom monitoring is especially relevant for affective disorders, in which reliable diagnostic and predictive biomarkers are still lacking and self-reported symptoms are not only influenced by recall bias, but also by a mood-state associated bias. Therefore, it is an important question whether long-term continuous monitoring of subjective and objective parameters combined with a time-series analysis could provide earlier and more reliable predictors and indicators of mood changes. Such findings could be helpful for the individual patient to improve their self-management and prevent relapses [23]. Although there is already an ever-growing number of systems (usually smartphone apps) focusing on monitoring depressive symptoms, as well as collection of objective data from smartphone or external sensors, only a small portion of these have been evaluated regarding feasibility, efficacy, and safety [23]. Considering the importance of this emerging field, the speed of innovations and new developments, it was our aim to review the state-of-the-art knowledge about smartphone-based monitoring systems for affective disorders and their benefits for treatment.

### Objective

The aim of this systematic review is to examine the literature on smartphone-based systems that combine subjective ratings with objectively measured data for the self-monitoring of depressive symptoms. The following aspects will be covered: (1) feasibility and adherence, (2) the association of monitoring data with mood status, and (3) effects on clinical outcome.

## Methods

### Overview

Literature was systematically reviewed in accordance with the Preferred Reporting Items for Systematic Review and Meta-Analysis (PRISMA) guidelines [30]. This systematic review has been registered in the PROSPERO international prospective register of systematic reviews (registration number CRD42017058539). A PRISMA checklist is presented in Multimedia Appendix 1.

### Information Source

In February 2017, the search for relevant articles published in the last ten years (2007-2017) was conducted in the following bibliographic databases: PubMed, Web of Science, PsycINFO, and Cochrane Central Register of Controlled Trials. Additional articles were identified via pearling, author correspondence, selected reference lists, and trial protocols.

### Search Strategy

The search strategy was developed by three of the authors and included combinations of the search terms, such as *affective disorder*, *ecological momentary assessment*, *smartphone*, *e-mental health*, and *self-monitoring*. The search took place in six content-related categories and the application of iterative combinations of these categories by employing the Boolean search operators (see Multimedia Appendix 2 for the full search strategy).

### Eligibility Criteria

Titles and abstracts were screened for eligibility and were included if: (1) abstracts were published in English or German, (2) any form of smartphone-assessed self-monitoring of depressive symptoms was mentioned, and (3) adult study participants with depressive symptoms (but not postpartum/postnatal depression or pregnant women) were investigated. In cases of insufficient information to determine eligibility, papers were nonetheless subjected to further screening. Considering the novelty of this research area, the review was planned to be as inclusive as possible. Therefore, experimental and observational studies were included.

### Selection of Studies

We obtained full reports of all potentially relevant papers. Articles were discarded if they met at least one of the following exclusion criteria: (1) review papers only; (2) study protocols or descriptive planned studies or future study intents; (3) descriptive studies that did not include participants diagnosed with affective disorders (apart from postpartum/postnatal depression); (4) studies that did not monitor objective data involving a smartphone/mobile device (eg, computerized/Internet-delivered or Internet-based Cognitive Behavioral Therapy interventions or Ecological Momentary Assessment [EMA] studies in which only subjective ratings were obtained); or (5) other reasons (eg, comments, letters, poster abstracts, articles not written in English). Additional information from study authors was obtained when necessary to resolve questions about eligibility. Four authors (ED, EK, CS, and XW) screened the full text reports and decided whether these papers met the inclusion criteria. Records were originally split among the four reviewers, each of whom made independent decisions regarding exclusion. Uncertainties and disagreement were resolved through discussion (ED, EK, CS, and UH). Reasons for excluding articles were recorded (including multiple assignments to exclusion criteria, if applicable). Finally, all exclusion assignments were reviewed and each record was allocated to one exclusion criteria (CS, XW, and EK).

### Assessment of Methodological Quality

The Downs and Black Instrument [31], which is recommended for both randomized and nonrandomized studies, was used for assessing the quality of included studies. This 27-item checklist rates studies on the following subscales: reporting, external validity, internal validity-bias, internal validity-confounding (selection bias), and power. The total maximum score that can be achieved with this instrument is 32. High internal consistency, as well as good inter-rater variability and test-retest-reliability, were reported [32]. For the purpose of this study, the last question was modified by changing the score from 0-5 to a dichotomous score (0/1) equivalent to the procedure outlined by Hootman et al [32]. If a sample size or power calculation was provided, the final item was scored as 1. The item was scored 0 if sample size and power calculations (or explanations about the appropriateness of the sample size) were missing. Thus, the total maximum score of this modified checklist is 28.

### Data Synthesis

Due to the novelty of the approach/research field and the heterogeneous nature of the included studies, a quantitative data synthesis (meta-analysis) has not been performed, since there was an insufficient amount of data to calculate effect sizes. Standardized forms were developed for data collection purposes and two authors (ED and EK) were trained to use and apply them. Piloting of the extraction forms with a subsample of randomly selected included studies also took place. To ensure consistency between reviewers, calibration exercises took place before starting the review. Data extraction was performed in duplicate by two authors (ED and EK), disagreement was resolved by discussion, and another author (CS) verified the information to reduce bias and errors in data extraction. We contacted study authors to resolve any uncertainties or for clarification, if needed. A systematic narrative synthesis will be provided with information presented in the text and tables to summarize and explain the characteristics and findings of the included studies.

## Results

After the description of the selection process and characteristics of included systems and studies are detailed, the results section is structured according to the objectives of the review. The *Association of Monitoring Data With Mood Status* section lists the different parameters recorded by the smartphone-based systems included in the review and, where applicable, is extended by further evidence.

**Figure 1:**
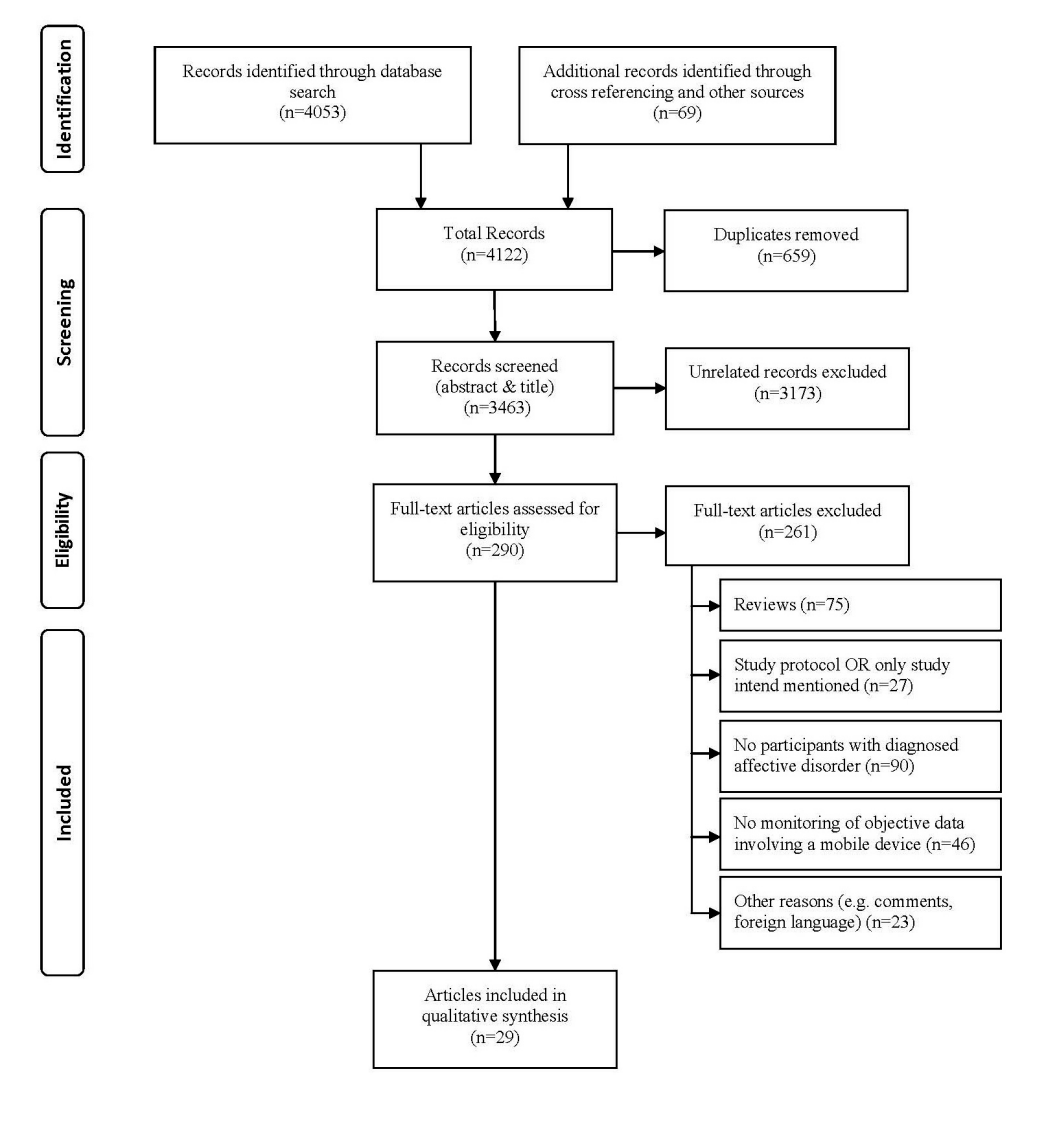
PRISMA Flow chart.

The electronic database search resulted in a total of 4122 records (PubMed, n=650; PsycINFO, n=378; Web of Science, n=1534; Cochrane Central Register of Controlled Trial, n=1491; and n=69 identifications by cross-referencing), of which 3463 were unique articles. In the first selection step (abstract screening), 3173 records were excluded after it was determined that they did not meet inclusion criteria based on information in the abstract. The majority of these studies were excluded because they only contained descriptions of online/computerized Cognitive Behavioral Therapy systems, descriptions of pure online interventions, or self-monitoring or screening tools (using only subjective assessment and self-monitoring or smartphone-based ecological momentary assessments). A total of 290 full text papers were retrieved for further consideration. After reviewing these full texts, an additional 261 records were excluded. Figure 1 depicts a flowchart of the screening process (see Multimedia Appendix 3 for the list of excluded full text articles).

### Characteristics of Included Systems and Studies

Twenty-nine studies were included for qualitative synthesis in this review. Although the search range spanned from 2007 to 2017, the included studies were all published after 2010, attesting to the novelty of the research field. Table 1 provides an overview of all systems that were described within the included articles. Most systems incorporated different data sources: in all cases, subjective ratings were required by the users (eg, ratings on mood, estimates on sleep, or information on medication). These data were complemented by different bio-parameters (eg, electrocardiogram [ECG], respiration, skin conductance), which were either recorded by the smartphone itself (eg, accelerometer, microphone) or by additional wearables. Smartphone usage was monitored by many of the included systems, although different parameters were used (eg, screen state, number of incoming and outgoing calls). Smartphone sensors also provided information about context and environmental conditions that were taken into account (eg, light sensor, GPS).

A detailed description of the included studies is provided in Multimedia Appendix 4. Studies were conducted in the following countries: United States/England (n=10), Austria/Germany (n=8), Denmark (n=8), and Italy/France (n=3). The majority of included studies used an observational design (n=27), as most were pilot or feasibility studies focusing on feasibility of, and adherence to, the respective monitoring systems, or were aimed at investigating the association of monitored data with clinical mood state. Only three studies [33-35] provided data on effects of monitoring on clinical outcomes; of those, only one used a randomized controlled trial (RCT) design [35]. Accordingly, the sample sizes ranged from 1 to 78. While 4 studies included patients with diagnosed major depression, 24 studies were conducted with patients with bipolar affective disorder. Two studies [36,37] also included schizophrenia patients in their study population with affective disorders.

**Table 1:** Overview of smartphone-based systems that combine subjective mood ratings and objective assessment of physiological and behavioral parameters for the monitoring of affective symptoms.

	Self-Report Data	Physiological Data	Behavioral Data			Context and Environmental Data		Type of clinical trials
			Phone Usage	Physical Activity	Voice features	Light Exposure	Location	
Empath [38]	+	+	-	+	+	-	-	Case Study
FINE [39]	+	-	+	+	-	-	+	Feasibility Study
MedLink [33]^a^	+	-	-	-	-	-	-	Pilot Study
Mobilyze! [34]	+	-	+	+	-	+	+	Feasibility Study
MONARCA [26,35,40-44]	+	-	+	+	+	+	+	Feasibility Study, Pilot Study, RCT
MoodRhythm [45]	+	-	+	+	-	+	-	Pilot Study
PSYCHE [46-48]	+	+	-	+	+	-	-	Pilot Study
SIMBA [49]	+	-	+	+	-	-	+	Pilot Study
Unnamed System [50]^b^	+	+	+	+	-	-	-	Feasibility Study
Unnamed System [36]	+	+^c^	-	+	-	-	-	Pilot Study

^a^Objective monitoring of medication adherence using a *Wisepill bottle*.

^b^System was utilized in an Automated Monitoring of Symptom Severity (AMoSS) study.

^c^An ingestion sensor was used for gathering the physiological data.

### Results on Methodological Quality Assessment

According the Downs and Black instrument, the methodological quality scores of the studies ranged from 5 [44] to 22 [35]. Methodological bias of the included studies is presented in Multimedia Appendix 5. In most cases the included studies had low scores, as they were mostly conducted as single-arm observational trials, feasibility studies, or pilot studies, and were not randomized trials. In addition, studies reporting only qualitative data [37,39,50,51] also scored lower. Except for one study [36], no adverse effects were reported in the selected studies.

### Results on Feasibility and Adherence

Among the included studies, large variations in drop-out rates were reported, ranging from 0% [26,33,39,44,46,48,50,52-56] to 22% [45]. Technical problems, including inappropriate operating systems [34,40,45,57], were reported to be the most common reason for discontinuation. Several articles reported on feasibility and discussed aspects of improving adherence. Prociow et al [51] reported large initial interest when approaching self-support groups and charities, but only three patients were willing to participate in their feasibility trial, of which two withdrew before a second visit. Compared to their healthy participants who were described as being “accustomed with mobile technology”, the patient (although being euthymic during the study duration) adhered only poorly to the monitoring regimen due to self-reported difficulties with the management of the unfamiliar technology. The system in this study was comprised of environmental as well as wearable sensors, with the latter causing a higher level of discomfort.

In a field trial of MONARCA 1.0 [40] the adherence rates for phone-based and paper-based self-assessments were found to be comparable. Moreover, high compliance scores of the four mandatory self-assessment parameters by phone-based self-assessment were reported. The patients in this field trial also rated the system usability and usefulness on a Likert scale from 1 (strongly agree) to 7 (strongly disagree). Average rates for these scores were: overall satisfaction=2.6, system usefulness=1.93, information quality=3.32, interface quality=2.71, usefulness of the system for disease management=3.16, and perceived usefulness for disease management=2.16. Taking into account the days on which the system was working, an adherence rate of 87% was reported for this field trial, with an increased adherence (91%) being reported for MONARCA 2.0 [44].

Beiwinkel et al [49] reported a compliance rate of 55.7% for filling in self-reporting data, with no decline over time, during an average study duration of 12 months. In a pilot study (n=28) assessing feasibility of a digital health feedback system using an ingestion sensor for physiologic assessments and confirmation of tablet ingestions, a medication adherence rate of 74% was documented [36]. Among the participants who completed the study (n=27), the concept was found easy to understand by 70%, 89% reported that the system might be useful for them, and 78% indicated that they would prefer to receive reminders on their smartphones in case of forgetting to take the medications [36]. In the pilot study (n=8) of the Medlink system [33], medication adherence was assessed through the *Wisepill bottle* which produces a signal when it is opened, thus providing real-time information about patient pill-taking activity. According to data gathered from the pillbox, 72.5-100% of doses were taken by study participants [33]. On a scale from 1 (strongly disagree) to 7 (strongly agree) the average rating for satisfaction was found to be 4.8 (standard deviation [SD]=0.8). Ease of use was rated 5.7 (SD=1.1), while learnability scored 6.1 (SD=1.5) and an average rate of 4.6 (SD=1.0) was found for usefulness.

The overall satisfaction of users with smartphone-based systems is reported to be rather positive (eg, the satisfaction with the Mobilyze! intervention was reported to be 5.71 [SD=1.38] on a scale ranging from 1 [strong disagreement] to 7 [strong agreement]) [34]. However, the usage pattern in this study showed a constant decline over time with participants using their phone 15.3 times (SD=8.3) during the first week of the intervention, 9.0 (SD=6.5) during the fourth week, but only 4.8 times (SD=4.6) during the eighth week [34]. In a post-study usability survey of MoodRhythm, the average System Usability Scale rating among participants was found to be 85.94 (SD=10.43) out of 100 [58].

Patient compliance was reported as a limitation in several studies [39,41-43,45,49,51,52,54,55,59], as it could not completely be controlled if participants carried their smartphone with them all the time, did not share their phone with others, or did not turn it off occasionally during the monitoring phase. In the study by Muaremi et al [60], 6 of 12 included participants were excluded for mood state classification analyses due to either not experiencing mood state changes, or data incompleteness caused by not using the study smartphones for phone-calls or switching it off for several days. Maxhuni et al [53] also used information from only 5 of 10 participants due to the lack of data gathered from phone calls. As a precaution, Faurholt-Jepsen et al [26] did not include participants who were not willing to use the study smartphone regularly.

Reporting experiences from a first field trial of their FINE app prototype, Dang et al [39] stated that patients provided information about using the study smartphone only irregularly. However, the patients shared that they would be interested in using the device in addition to their conventional treatment if transparency concerning the recorded data and its further use was given [39]. Participants also criticized not being allowed to disable the background data logging, specifically mentioning GPS tracking [39]. Saunders et al [50] interviewed 21 patients that had participated in a self-monitoring study and found that patients considered it very important that assessment devices look normal to avoid drawing attention in public. Wrist-worn actigraphy devices and a daily mood rating were considered tolerable; a higher frequency of assessments (in the study, up to 10 mood samples during a day were collected) was considered inconvenient [50]. All but one patient was willing to share their data with others, especially since most considered this to be beneficial for the decision making of their psychiatrists; however, patients also stated that they would be interested in giving context explanations to their data to avoid misinterpretations [50]. The size of wrist-worn actigraphy devices was also rated favorably by the participants of the Naslund et al study [37]. Wearing the device was considered uncomplicated, yet some participants with limited prior experience with mobile technology experienced operating with the unfamiliar smartphone technology as distracting and discouraging [37].

### Results on Association of Monitored Data With Mood Status

At present, most empirical data exist on the association between objectively recorded data and clinical state or symptom changes (self-reported or rated by clinicians). Therefore, the following section summarizes results obtained with the different (bio-) parameters. If applicable and relevant, additional evidence from nonincluded publications is given to provide a comprehensive overview. Based on our results, no cumulative statements about the direction and/or magnitude of effect sizes can be drawn, since all studies differed concerning selected features, statistical approaches, and handling of ground truth (classification of affective states) for mood state prediction. Overall, the prediction accuracy depended largely on the methodological approach and the respective affective states as outcome measures (eg, depression versus euthymic state compared to depression versus [hypo]mania).

Many of the included studies reported results on a small number of subjects. However, when individual time-series are to be analyzed, a sufficient recording duration for the individual patients seems to be more desirable than a larger number of subjects. Twenty-five studies monitored data for several months or weeks, three of which still had to exclude subjects from the final analysis, as they did not experience any changes in mood states, and thus associations between mood states and objective parameters could not be analyzed [54,55,60].

#### Self-Assessment/Self-Report

Self-assessment or subjective rating of mood status via different assessment methods is an integral part of nearly all included studies and systems currently being tested. The superiority of EMAs in mental health versus standard paper-pencil questionnaires has been proved by many studies [61]. Among the selected studies, 20 [26,33-36,38-45,49,50,52,53,55,57,60] provided a self-assessment option. Mood agenda, sleep agenda, and diary have also been included in the PSYCHE platform [46], but no findings were reported in that context.

The content of self-monitored items varies from system to system. Within the MONARCA system, self-assessment of mood, sleep length, medication adherence, irritability, activity, mixed mood, cognitive problems, stress, alcohol consumption, menstruation for women, and individualized early warning signs are requested [62]. In the system used by Grünerbl et al [57], information about the activities of daily living as well as psychological state, physical state, and amount of activity were assessed daily. Within the SIMBA system, there were only two questions about mood and energy [49]. Several systems use established questionnaires. In the MoodRhythm system the Social Rhythm Metric (SRM-5) is used [45]; the FINE app monitors mood through administration of the Patient Health Questionnaire (PHQ-9) [39], and within the MedLink system, mood was assessed with PHQ-9 [33]. The Center for Epidemiologic Studies Depression Scale is administered weekly in the Empath system [38], and in the system used for the AMoSS study *Mood Zoom* was used for daily mood monitoring while the *True Colours* system was selected for weekly mood measurements [50]. In the pilot study of a digital health feedback system using an ingestion sensor for confirmation of medication ingestion and measurement of physical metrics, a self-assessed sleep quality questionnaire was used [36]. Additionally, in some systems there are extra self-assessment opportunities, such as notes or diary sections that are incorporated [45]. However, in the field trial of MONARCA 1.0 [40] it was reported that participants did not use the optional text entry on the phone.

Self-assessments may serve different purposes: they provide a continuous support to individuals (eg, supportive monitoring) and also allow for a better estimation of individuals’ need for support, and may serve as an underlying information system to guide and tailor care accordingly (eg, by detecting symptom deterioration or early warning signs for change in moods states) [63]. This longitudinal technology-enhanced assessment of symptoms and behaviors (eg, outcome monitoring and EMA) can help to improve our understanding of illness development and recovery, and can further optimize care by integrating self-monitoring systems into specific e-mental health interventions and apps. In the study of Mobilyze! [34], self-reported items were used for individualizing prediction models. Frost et al [44] showed in the field trial of MONARCA 2.0 that self-reported activity, stress, and sleep were among the three ranked parameters according to the correlation between Impact Factor and the mood score. Faurholt-Jepsen et al [26] showed that there was a significant correlation between Hamilton Depression Rating Scale, 17 item version (HDRS-17) scores and self-rated mood in adjusted (for age and sex) and unadjusted models. Another study by Faurholt-Jepsen et al [43] showed significant positive correlations between self-monitored mood rate and HDRS-17 in adjusted and unadjusted models (n=29). In relation to manic symptoms, the Young Mania Rating Scale (YMRS) correlated significantly and positively with self-monitored data on mood in adjusted and unadjusted models. Maxhuni et al [53] reported that adding information from questionnaires to the information from accelerometer and audio features improved the overall accuracy results of classifying bipolar disorder episodes; however, they emphasized that precision and recall results were very similar with and without questionnaires.

#### Physiological Parameters

According to Valenza et al [64], studying mood swings over time shows that changes in mental status have intrinsic dynamics, which suggests a link between autonomous nervous system dynamics (a division of peripheral nervous system) and bipolar disorders. These changes can be shown in physiological parameters such as respiration activity, heart rate variability (HRV), and electrodermal activity. Physiological parameters were assessed in four of the included studies [36,46,48,50]. Since two of the studies did not report any association with the mood states, only the other two will be discussed.

Using the PSYCHE platform, ECG/HRV and respiration activity were gathered using a sensorized t-shirt [46]. Additionally, an accelerometer for measuring physical activity was included in the wearable system. The collected raw data was preprocessed and feature extraction was performed to determine data series for the HRV and respiratory rates. Studies using the PSYCHE platform to identify and predict mood states indicated that HRV series can be employed for classification of mood states: after analyzing HRV series Lanata et al [46] suggested that sample entropy of these series can be regarded as a marker for clinical severity in bipolar disorders. In a study analyzing long-term HRV dynamics in eight patients with bipolar disorder, Gentili et al [48] found that HRV features that were normalized using information from future and past mood states provided a significantly higher classification accuracy (mean=99.52) compared with other HRV-features, which were normalized using other procedures or were not normalized at all. The authors claimed that the results of this study show the high accuracy of HRV features in labeling of mood states [48].

#### Phone Usage

During a depressive phase, the desire and ability for social interaction is mostly reduced, whereas during the (hypo)manic phase it is increased [59]. The included studies measured social interaction through duration and number of incoming and outgoing phone calls and number of incoming and outgoing short messaging system (SMS) texts per day as a behavioral marker. Nine of the included studies [26,34,42,43,49,52,57,59,60] reported the association between social interactions with mood states.

According to Grunerbl et al [57] the length and number of phone calls increase in mild depressive phases compared to severe depression or a normal mood state. In another study by the same group [59], it was reported that behavioral data only from phone call features did not provide high recognition rate. Muaremi et al [60] examined phone call features in three groups: phone call statistics (eg, number of calls/day), social signal processing (eg, average speaking length), and acoustic emotion recognition (eg, pitch frequency). The average mood state prediction performance of phone call statistics was lower than other features [60].

Within the MONARCA project, significant positive correlations between the number and duration of incoming and outgoing calls per day and scores on YMRS, and between scores on the HDRS-17 and duration of incoming and outgoing calls per day, were found; additionally, a significant positive correlation between scores on the YMRS and the number of outgoing text messages per day was shown [42]. In another study from this group [26] with bipolar patients (n=17) there was no significant correlation between affective symptoms assessed with HDRS-17 and YMRS and the number of outgoing text messages. In another study with 29 participants [43] HDRS-17 scores were found to be significantly and negatively correlated with numbers of incoming text messages per day as well as numbers of outgoing calls per day, whereas positive correlations were shown between numbers of incoming calls and missed calls per day and HDRS-17 scores. In relation to manic symptoms, YMRS scores were found to be significantly and positively correlated with numbers of outgoing text messages per day and daily duration of phone calls in adjusted models [43]. Furthermore, daily duration of outgoing calls, as well as number of characters in incoming text messages, were significantly and negatively correlated with YMRS scores [43].

Beiwinkel et al [49] found a negative association between number of outgoing SMS texts and HDRS-17 scores in within-patient analyses and between-patient analyses. As duration of calls did not show any significant relationships with YMRS and HDRS, the number of calls made was positively associated with manic symptoms, assessed via YMRS in between-patient analyses [49].

Phone usage patterns including screen state and usage of apps might provide information about patients’ current mood state. Alvarez-Lorenzo et al [52] reported that self-reported mood level showed a negative correlation with the amount of time the phone screen is on, the percentage of social apps, the percentage of entertainment apps, as well as the amount of time patients interact with the smartphone, respectively (n=18). Conversely, positive correlations between average number of apps and self-reported mood level, as well as between browser apps (eg, Chrome, Firefox) and self-reported mood level, were reported [52]. For 17 bipolar patients, no significant correlation between the amount of time the screen was on and affective symptoms (assessed with HDRS-17 and YMRS) was reported by Faurholt-Jepsen et al [26]. However, in another study by this group [43], duration of screen-on per day was found to be positively and significantly correlated with HDRS-17 scores in unadjusted as well as adjusted models.

#### Physical Activity

Patients with affective disorder tend to have lower acceleration and activity energy expenditure compared to healthy individuals [65]. Furthermore, a lower activity energy expenditure per day and acceleration have been demonstrated in bipolar patients compared to patients with unipolar depression [65]. By gathering the physical activity data continuously, it is possible to observe the effects of physical activity on mental health more comprehensively. Among the included studies, physical activity was measured mainly with accelerometers incorporated in the smartphones. In the PSYCHE project [46], the accelerometer was integrated into a wearable system.

Eight of the selected studies reported on the relationship between affective states and physical activity [38,45,49,53-55,57,59]. In a study with 12 bipolar patients, the average precision/recall values (for state change detection) were approximately 60% for acceleration [55]. Conversely, in a study with 10 bipolar patients an average recognition accuracy of 70% for movement features (measured with accelerometers) was reported by Grünerbl et al [59]. This finding was supported by results from an older study by this group [57], in which an increase in motion ratio occurred with improved mood state. Furthermore, the authors pointed out that in manic patients the trend was reversed: with the progression from manic to normal state, a decrease of 33.7% in the amount of movement was detected [57]. Abdullah et al [45] pointed out that nonsedentary duration was weakly correlated with mood pattern. Beiwinkel et al [49] reported that device activity (measured by accelerometers) did not have a significant relationship with clinical symptoms in between-patient analyses. Osmani et al [54] reported a correlation between the overall physical activity level and the patients’ state. Furthermore, this study found a much stronger correlation between the physical activity divided in daily intervals and patient’s state than there was for the overall physical activity levels [54]. Maxhuni et al [53] reported that using accelerometer features recorded during telephone conversations have accuracy results over 80% for classifying bipolar disorder episodes. According to these analyses, the frequency domain feature showed better performance in comparison to the time domain feature [53]. However, Dickerson et al [38] could not find a significant relationship between mood levels and a movement factor.

#### Location

Changes in travel patterns (eg, distance travelled per day, location changes), which can be gathered through GPS, Bluetooth (via detection of other wireless devices), cell tower identification (ID) and WiFi, may provide information about patients’ mood states. For example, it is expected that people in a depressive phase travel and stay outside less often [55]. Eight studies [26,34,43,45,49,55,57,59] investigated the association between travel patterns and mood states. Promising accuracy rates for state recognition by using travel patterns were shown in two studies [55,59]. Grünerbl et al [57] found an increase of 200% in the average time spent outside from a depressive state to normal state. Beiwinkel et al [49] reported that the amount of movement between cell towers (indicating changes of location) did not have a significant relationship with clinical symptoms but distance travelled showed significant negative association with clinical manic symptoms in between-patient analyses. Additionally, in within-patient analyses a negative relationship between the increase in cell tower movements and clinical manic (as well as depressive) symptoms was found [49]. Conversely, changes in distance travelled were not significantly related to symptom change [49]. Faurholt-Jepsen et al [26] reported a significant correlation between the higher score on HDRS-17 and lowered number of changes in cell tower ID per day in their unadjusted model; however, in their adjusted model the correlation was borderline significant. In another study using MONARCA system [43], Faurholt-Jepsen et al reported significant positive correlations between cell tower ID changes per day and both rating scale scores (YMRS and HDRS-17). In the study by Abdullah et al [45], a significant positive correlation between location clusters and trend of self-assessed energy scores was found, while location clusters had the second highest ranking among distance travelled, nonsedentary duration, and conversation frequency in stable and unstable status classification.

#### Light Exposure

Light density in the environment can be used to provide information about patients’ sleep-wake cycles, which is disturbed in unipolar depression as well as in bipolar disorder [66,67]. Differentiation between natural and artificial light can also be used to approximate time spent outdoors, which has also been shown to have a negative correlation with depressive symptoms [68]. Only three of the included studies [34,45,51] used ambient light sensors. Prociow et al [51] added a light detector as an environmental sensor in their system. The Mobilyze! app also used ambient light data to approximate environmental context, but technical problems were reported (implausible results above the maximum meaningful value were obtained by the ambient light sensor) [34]. The MoodRhythm system included light sensor data in its evaluation of sleep-wake cycles [58], while the study protocol of the MONARCA 2.0 trial categorized ambient light under phone usage information [62]. The monitoring of ambient light is also planned in the further development of the PSYCHE platform [69].

#### Voice Features

Analysis of speech and voice features appears to be a useful approach in predicting mood state in unipolar depression and in bipolar disorder [70,71]. Furthermore, using microphones to capture ambient sound can allow for the gathering of the number of conversations as a measure of social isolation, which is a behavioral pattern common in depression [45,59].

Eight articles [38,41,45,47,53,56,60,72] reported on relationships between voice features and affective states. The study by Gideon et al [72] dealt with a typical challenge of real-life monitoring of speech patterns: patients use different smartphones causing acoustic variations during the voice recordings. Using various approaches for preprocessing, feature extraction, and data modeling, the authors were able to increase the discriminative power of their mood state prediction algorithms [72]. However, only structured interview calls were analyzed in this study. Karam et al [56] also demonstrated that mood states of bipolar individuals can be separated based on voice recordings from clinical interactions and that hypomania can also be detected from unstructured calls, whereas their system struggled to detect depression from voice recordings during nonclinical interactions. Abdullah et al [45] reported that conversation frequency was weakly correlated with mood pattern. Within PSYCHE project, Guidi et al [47] concluded from a case report that changes in voice pitch features can be extracted via smartphones and correlate significantly with changes between euthymic, depressed, and hypomanic states in a bipolar patient.

Analyses of voice and speech features are also performed within the frame of the MONARCA project. Muaremi et al [60] extracted three types of features from phone call conversations and reported that using acoustic features (eg, kurtosis energy, pitch frequency) resulted in the best state recognition in comparison to phone call statistics (eg, number of phone calls during the day, average duration of phone calls) and social signals (eg, average speaking turn duration, average number of speaker turns). Maxhuni et al [53] reported that using audio features (emotional and spectral) resulted in accuracy results >80% for classifying the mood state of patients with bipolar affective disorder. Testing emotional and spectral features isolated and together resulted in similar accuracy [53]. Faurholt-Jepsen et al [41] reported an accuracy ranging from 0.61 to 0.74 in the classification of mood states based on voice feature. Dickerson et al [38] found speech factors and sleep factors more indicative of depression than other features measured in this case study.

#### Aggregation of the Data Collected From Smartphone Sensors

Most of the reviewed papers described a combination of self-reported data with behavioral and/or physiological parameters. Grünerbl et al [55,59] reported that the combination of features (eg, social behavior, travel patterns, and movement) optimized the state detection and provided better results than single sensors or fusion of only a few sensor modalities. Abdullah et al [45] also reported that combining self-reported data with data from several smartphone sensors (light sensor, accelerometer, microphone) and communication patterns resulted in reliable prediction of SRM-5 scores (75.89% of correctly classified labels had a probability >.7). Ranking the used features according to their importance for the classification of stable and unstable status (according to SRM-5 scores) showed the following order: distance travelled, location cluster, nonsedentary duration, and finally conversation frequency. Maxhuni et al [53] found only a small improvement in average classification accuracy for emotional states of their participants when audio features derived from phone conversations were combined with accelerometer features recorded during phone conversations. Faurholt-Jepsen et al [41] stated that increases in the sensitivity, specificity, and accuracy were found after combining voice features, self-monitored data, and automatically generated objective data. The Empath system described by Dickerson et al [38] also aims at combining several behavioral markers into a depression index, or a global depression risk index. However, only a single patient case study was reported, mainly dealing with feasibility issues [38]. Prociow et al [51] also introduced a system that combines environmental (motion detectors, light detector, microphone, door and bed sensors, remote control monitor) and wearable sensors (accelerometer, light detector, microphone, GPS).

### Results on Effects on Clinical Outcome

Most of the reviewed studies did not report any results regarding the effects of smartphone-based monitoring on depressive symptoms or clinical outcomes. However, Burns et al [34] reported that during the Mobilyze! intervention program, depressive symptoms (self-reported on the PHQ-9) decreased significantly over time (t_13_=7.02, beta_week_=–.82, *P*<.001) according to intent-to-treat analyses. Moreover, this study found that evaluator-rated depressive symptoms (rated on the Quick Inventory of Depressive Symptomatology) improved (t_13_=8.22, beta_week_=–.81, *P*<.001) and it became less likely for participants to fulfill diagnostic criteria for Major Depressive Disorder (Z=2.15, beta_week_=–.65, *P*=.03) [34]. Additionally, anxiety symptoms decreased (t_13_=4.59, beta_week_=–.71, *P*<.001) according to Generalized Anxiety Disorder questionnaire scores [34].

Saunders et al [50] performed qualitative interviews with a subset of participants (n=21) of the AMoSS study, which had monitored their moods using a smartphone combined with movement sensing devices. The authors reported that most patients (n=16) experienced the self-monitoring as enhancing their illness insight and that half of their patients stated that they had improved their mood due to being able to better identify their feelings [50]. Half of the patients also reported a change in behavior, in most cases related to increased exercise levels, but four patients also pointed to potential negative effects due to becoming too occupied with the monitoring [50]. Naslund et al [37] provided 11 patients with activity tracking devices combined with smartphones for a 6-month lifestyle intervention aimed at weight reduction, and then performed qualitative interviews. Participants considered the devices to be motivating and to increase awareness about their level of physical activity, thus helping them to increase their overall activity [37]. In the pilot trial of MedLink system [33] which assessed medication adherence through a *Wisepill bottle*, a decrease of depressive symptoms assessed with PHQ-9 was observed (*P*=.008). The authors suggested that lag time between monitoring and the initiation of medication can be reduced via the MedLink system [33].

Results from only one RCT were available. Faurholt-Jepsen et al [35] reported that in the MONARCA 1.0 trial patients in the intervention group showed more depressive symptoms during the trial according to HDRS-17 scores compared with the control group (B=2.02, 95% CI –.13 to 4.17, *P*=.07). Additionally, in further analyses (excluding mixed depressive and manic symptoms in both adjusted and unadjusted models) significantly more depressive symptoms were reported in the intervention group [35]. No difference in manic symptoms (based on the YMRS) was observed between the intervention and control groups (B=–.34, 95% CI –1.14 to .47, *P*=.41). However, excluding mixed depressive and manic symptoms, a trend for fewer manic symptoms in the intervention group was reported compared to the control group in the adjusted model (B=–1.07, 95% CI –2.15 to 0.005, *P*=.051) [35]. Furthermore, a trend towards a higher score of subjectively perceived stress was reported in the intervention group compared to the participants in the control group (adjusted model: B 2.40, 95% CI -.33 to 5.13, *P*=.09). No differences in psychosocial functioning, quality of life, self-rated depressive or manic symptoms, or self-rated or screened cognitive function were found between the intervention and control groups [35].

## Discussion

To our knowledge, this is the first systematic review of the current scientific literature on smartphone-based systems for the subjective and objective monitoring of mood disorders and studies testing these systems. A considerable number of projects have started developing smartphone-based tools for the management and monitoring of mood disorders and have conducted promising tests of feasibility. Nonetheless, it became obvious during the review process that only a small number of systems that are currently available (or in preparation) have been described in scientific papers, let alone been subjected to empirical studies.

The results showed great potential in monitoring parameters relevant to the course of affective disorders, although most of the reviewed studies were feasibility tests or pilot trials without control groups, conducted with only a small number of participants and with short assessment periods, and did not investigating any medium- or long-term effects or possible risks of the respective applications. It is important to highlight that there are several systems described in publications that could not be included in this review due to: not meeting the inclusion criteria (eg, no monitoring of objective data [73,74]), not involving a smartphone for the objective monitoring [64,69,75-77], not having studied participants with an affective disorder diagnosis [78-81], or not having published results from trials being conducted at the time of our search [82].

Given the lack of reliable biomarkers in affective disorders, the combination of different physiological and behavioral parameters seems to be a highly promising approach [83,84]. This is especially true if time-series analyses are applied to continuously monitored behavioral and/or physiological data from individual patients to predict changes in their clinical states. It can be noted that significant heterogeneity exists with respect to how the data was analyzed across studies. Many groups developed their own data analytic approaches (eg, machine learning algorithms) in parallel to the set-up of their systems, and the chosen approaches have considerable impacts on the results of the respective studies. Those studies using such approaches could show good prediction values and probability estimations for changes in mood states or clinical outcomes [45,55,59].

The acceptance of using smartphones for continuous monitoring (of subjective and objective data) seems encouraging based on the results of this review and reflects patients’ interests. However, regarding the decrease in adherence rates in several studies [34,78], some caution might be taken to ensure the continuity of usage. For instance, a promising concept for user engagement is the employment of gamification (game design elements such as infographics, feedback, activity, and progress bars [85]). Future trials should use reminders and/or other evidence-based patient engagement methods (eg, financial incentives, gamification, and peer mentoring) to minimize the problem of missing data that stems from nonadherence of carrying or using the smartphone for data collection.

Technical issues that might occur in this context (ie, energy management) could serve as a major obstacle. Using external sensors that do not rely on the smartphone’s power source was discussed as one possible solution, but Prociow et al [51] reported that any additional devices were perceived as uncomfortable by the patients and tends to increase reluctance to use the system. It is also important to enable data collection from as many operating system as possible, since using another smartphone system was reported to be a reason to decline the participation of a study [41,43].

Only five of the reviewed articles reported on clinical outcomes [33-35,37,50]. In the pilot study of the Mobilyze! intervention program [34] the depressive symptoms were decreased during the trial. Conversely in the first RCT of the MONARCA system [35] the exploratory analyses in relation to primary outcomes revealed that electronic daily self-monitoring (including feedback to the users as well as their health care practitioners) may improve manic symptoms but also sustain depressive symptoms among some, but not all, patients. Following the authors’ explanation, it can be speculated that self-monitoring increases illness insight and self-awareness, which are often lacking in phases of (hypo)mania. Therefore, patients within this mood state might benefit more than depressed patients, where a daily confrontation with their perceived shortcomings might sustain or even worsen their depression. However, the potential risks and adverse effects associated with continuous self-monitoring should be considered and carefully evaluated. Surprisingly, these aspects are highly underrepresented or even neglected in the recent literature body. Conceivably, a fragmentation of daily life, increased somatization due to continuous monitoring of biological parameters, negative effects on attention, decreased mindfulness, and social and behavioral aspects could be potential negative effects [86,87]. These considerations indicate the importance of conducting more experimental and randomized trials with larger samples sizes and longer assessment durations.

Combining smartphone-based self-monitoring with digital intervention strategies has a high potential for improving the current treatment and management of affective disorders. By investigating the efficacy of smartphone-based interventions, the notion of the “digital placebo” effect [88] should also be taken into account. High levels of expectation, trust, and personalization of smartphones influence the outcome of app-based interventions, as simply interacting with and receiving feedback from a smartphone app may already increase subjective well-being [88,89]. For example, one of the first studies on mental health apps by Kauer et al [90] showed that depressive symptom recording and self-monitoring via smartphone significantly reduced symptoms even without any direct therapeutic intervention. Not only the placebo effects, but also the adverse effects and safety issues of using digital mental health apps, are very rarely taken into account. A number of adverse effects that are unique to the use of mobile apps and the Internet have been discussed (eg, increased levels of inactivity, sleep pattern disturbances, eye strain, and reduced social skills [87]), but to date there is no information available on occurrence or frequency of risks, or whether certain mental health apps have a higher or lower risk potential than others.

Additionally, there are several ethical issues arising from continuous monitoring and the use of objective smartphone data. While some users/patients may find that remote monitoring allows them to feel more control over their illness, also opening new possibilities for patient-empowerment and self-management, others may feel overwhelmed by the added responsibility of self-monitoring, or perceive it as a constant reminder that they are ill [91]. The feeling of being watched or being under surveillance may also cause unease or even increase anxiety. Furthermore, there is a certain risk for patients and health care providers alike of placing too much trust in smartphone-based monitoring systems, especially concerning the (limited) possibilities of an adequate response in emergency situations (eg, acute suicidal ideation). The increasing usage and application of these technologies in mental health care and research poses the question of responsibility, and new ethical dilemmas will undoubtedly arise [92].

Sensitive data concerning mental health require an especially high standard of protection from security breaches due to a high risk of stigmatization and discrimination in the case of data disclosure. Patients’ concerns about the security of the data monitored and recorded by the system have significant impacts on the acceptance of the apps and the readiness to use them for continuous monitoring [93]. Processing data locally on the smartphone has been suggested as one possible solution [47]. The classification of mental health apps as medical products is also of capital importance for patient safety and acceptance. When a product is considered a medical product, the respective mental-health apps (especially apps focusing on specific disorders and symptom reduction) would need to meet certain quality standards, and would have to undergo efficacy and safety trials (comparable to the approval process of drugs or physical therapies). For example, this would ensure that patients would not falsely rely on a completely ineffective app or even use an app giving them potentially harmful recommendations [89]. However, not all of the available mental health apps (especially those focusing on self-management and lifestyle) are considered medical products, and therefore do not require these defined standards. To summarize, further attention should be given to questions of data security and confidentiality, especially regarding the huge amounts of mental health apps being openly available (eg, Apple App Store, Google Play Store) that show major flaws in security settings [94].

### Future Directions/Perspectives

Smartphone-based systems for managing and monitoring mood disorders present a highly promising field of innovation in health care. Simply using and carrying a smartphone on a day-to-day basis generates a larger amount of data than is typically collected in questionnaire-based studies or online interventions. Analyses of covariations of recorded bio-parameters within individual time-series and identification of individual patterns that predict state changes must be employed, in addition to group statistics. However, to identify such individual patterns, long monitoring durations with a certain amount of state changes are required. Most of the available studies used short or moderately sized recording durations and three of them [54,55,60] had to exclude participants from the final analyses, as they did not exhibit recognizable changes in mood state.

Apart from the continuous assessment of bio-parameters themselves, smartphone-based monitoring also allows researchers to gather information on context and environment, which could prove valuable for the interpretation of the monitored biomedical data (eg, information about weather conditions) and allow for a better interpretation of changes in body temperature or skin conductance levels (as well as locomotor activity) that might be restricted on rainy versus sunny days. Furthermore, these large amounts of data allow for the employment of data mining techniques to potentially generate and explore new hypotheses about causes and predictors of affective episodes, define individual disorder phenotypes, or analyze the consequences of changes in medication or treatment regimens, or potentially even health policy changes [94].

Continuous measurement of physiological and behavioral parameters is a valuable addition to self-reported symptoms to help complete the clinical picture, especially since there is often significant variation between measured parameters and subjective perspective of the self-reporting patient [95]. When considering disorders with impaired insight into illness, such as bipolar disorder [96], objectively measured data may provide a valuable method of tracking and predicting mood state changes. A highly valuable aspect of continuous physiological and behavioral monitoring approaches is the versatility of the usage of the resulting data: it could be used by the patients themselves to enhance self-monitoring practices and improve self-management strategies, and help clinicians adjust treatment by providing a more detailed picture of symptom trajectory. However, as of now, there is only sparse empirical evidence backing up these expectations, as most systems are currently in development and/or have only been studied within feasibility or pilot studies.

Smartphone-based monitoring systems can also be combined with electronically delivered psychotherapeutic interventions. By linking behavioral monitoring and intervention feedback loops, interventions can be delivered right-on-time at the most useful moment, in contrast to face-to-face psychotherapy mainly based on retrospective data and information [23]. However, fully relying on mental-health apps for disorder management and therapy would be placing false confidence and trust in a young technology. A broader empirical database is needed regarding effectiveness and potential adverse effects of continuous monitoring of behavioral and physiological data using smartphones. Meanwhile, an integrated treatment approach (face-to-face interaction between patients and clinicians and smartphone-based disorder management) should be preferred.

### Limitations

In this review, we have presented the current state of research on smartphone-based mental-health technologies for monitoring and managing mood disorders. A limitation of this publication is that it only covers those smartphone-based systems in which studies have been conducted, and results of these studies have been published in scientific literature. We also focused on articles listed in scientific literature databases; thus, grey literature (eg, conference proceedings and abstracts) was not included. There are a great number of apps for the monitoring and management of mood disorders available to the public that have either not been subjected to a scientific study, or the results have not been published (yet). Furthermore, some publications had to be excluded due to not reporting results on patients with affective disorders, although they were also introducing smartphone-based systems for affective symptoms. Another limitation is that a number of the included studies were feasibility studies, allowing conclusions to be drawn about the practicality of a certain monitoring technology, yet not about its clinical efficacy or effectiveness. However, the results of this systematic review on the current state of smartphone-based monitoring of depressive symptoms demonstrates that using smartphone apps for EMA and management of mood disorders is a promising field, in which more and larger efficacy studies and longitudinal research regarding placebo effects, possible risks, and adverse effects are required.

### Conclusion

Smartphone-based monitoring of objective and subjective data in mood disorders is a rapidly growing approach and research field. The current body of literature consists mainly of observational studies and substantiates the value of smartphone-based approaches for gathering long-term objective data (aside from self-ratings) to monitor clinical symptoms, predict changes in clinical states, and investigate causal inferences about state changes in patients with affective disorders. Although promising, a much larger evidence-base is necessary to fully assess the potential, as well as the risks, of these approaches. Fortunately, a number of study protocols stated ambitions to expand and intensify research in this emerging and promising field in the coming years.

## Appendix

Multimedia Appendix 1 PRISMA checklist.

Multimedia Appendix 2 Search strategy.

Multimedia Appendix 3 Reference list of excluded full text articles with reason for exclusion.

Multimedia Appendix 4 Overview of selected studies.

Multimedia Appendix 5 Methodological Quality Scores.

## Abbreviations

AMoSS: Automated Monitoring of Symptom Severity
ECG: electrocardiogram
EMA: Ecological Momentary Assessment
E-mental health: electronic mental health
GPS: global positioning system
HDRS-17: Hamilton Depression Rating Scale (17 item version)
HRV: heart rate variability
ID: identification
PHQ-9: Patient Health Questionnaire
PRISMA: Preferred Reporting Items for Systematic Review and Meta-Analysis
RCT: randomized controlled trial
SD: standard deviation
SMS: short messaging system
SRM-5: Social Rhythm Metric
YMRS: Young Mania Rating Scale
