# Supplementary material for: Smartphone-Based Monitoring of Objective and Subjective Data in Affective Disorders: Where Are We and Where Are We Going? Systematic Review
Source: J Med Internet Res. 2017 Jul 24;19(7):e262. doi: 10.2196/jmir.7006 (PMC5547249; doi:10.2196/jmir.7006)
Supplement: Multimedia Appendix 2 [file jmir_v19i7e262_app2.pdf]

Multimedia Appendix 2

Search strategy

1	(depress* OR<br>(mood disorder OR (mood AND disorder) OR mood disorder*) OR<br>(affective disorder OR (affective AND disorder) OR affective disorder*) OR<br>(bipolar disorder OR (bipolar AND disorder) OR bipolar disorder*))	
	A	(cell phone OR<br>mobile phone OR<br>(smart phone OR smartphone OR smart-phone))
	B	(self-monitor* OR (self-assessment OR self-assess*) OR physiological monitor OR personal monitoring system OR activity tracker OR track* OR screen* OR monitor* OR ecologial momentary assessment)
	C	(Telemedicine OR<br>(ehealth OR E-health OR e mental health OR e-mental health) OR<br>(mhealth OR M-health))
	D	(wearable* OR<br>(biosensor OR biosensor* OR bio-sensor OR bio-sensor* OR biomedical sensor OR biomedical sensor*))
	E	(prosod* OR<br>(voice analysis OR voice analyses OR voice analys*) OR<br>(speech analysis OR speech analyses OR speech analys*) OR<br>(phonetic analysis OR phonetic analyses OR phonetic analys*))

(((((((((((depress* OR (mood disorder OR (mood AND disorder) OR mood disorder*) OR (affective disorder OR (affective AND disorder) OR affective disorder*) OR (bipolar disorder OR (bipolar AND disorder) OR bipolar disorder*)))) AND ((cell phone OR mobile phone OR (smart phone OR smartphone OR smart-phone)))) AND ((self-monitor* OR (self-assessment OR self-assess*) OR physiological monitor OR personal monitoring system OR activity tracker OR track* OR screen* OR monitor* OR ecologial momentary assessment)))) OR (((((depress* OR (mood disorder OR (mood AND disorder) OR mood disorder*) OR (affective disorder OR (affective AND disorder) OR affective disorder*) OR (bipolar disorder OR (bipolar AND disorder) OR bipolar disorder*)))) AND ((cell phone OR mobile phone OR (smart phone OR smartphone OR smart-phone)))) AND ((Telemedicine OR (ehealth OR E-health OR e mental health OR e-mental health) OR (mhealth OR M-health))))) OR (((((depress* OR (mood disorder OR (mood AND disorder) OR mood disorder*) OR (affective disorder OR (affective AND disorder) OR affective disorder*) OR (bipolar disorder OR (bipolar AND disorder) OR bipolar disorder*)))) AND ((cell phone OR mobile phone OR (smart phone OR smartphone OR smart-phone)))) AND ((wearable* OR (biosensor OR biosensor* OR bio-sensor OR bio-sensor* OR biomedical sensor OR biomedical sensor*))))) OR (((((depress* OR (mood disorder OR (mood AND disorder) OR mood disorder*) OR (affective disorder OR (affective AND disorder) OR affective disorder*) OR (bipolar disorder OR (bipolar AND disorder) OR bipolar disorder*)))) AND ((cell phone OR mobile phone OR (smart phone OR smartphone OR smart-phone)))) AND ((prosod* OR (voice analysis OR voice analyses OR voice analys*) OR (speech analysis OR speech analyses OR speech analys*) OR (phonetic analysis OR phonetic analyses OR phonetic analys*))))) OR (((((depress* OR (mood disorder OR (mood AND disorder) OR mood disorder*) OR (affective disorder OR (affective AND disorder) OR affective disorder*) OR (bipolar disorder OR (bipolar AND disorder) OR bipolar disorder*)))) AND ((self-monitor* OR (self-assessment OR self-assess*) OR physiological monitor OR personal monitoring system OR activity tracker OR track* OR screen* OR monitor* OR ecologial momentary assessment))) AND ((Telemedicine OR (ehealth OR E-health OR e mental health OR e-mental health) OR (mhealth OR M-health))))) OR (((((depress* OR (mood disorder OR (mood AND

disorder) OR mood disorder*) OR (affective disorder OR (affective AND disorder) OR affective disorder*) OR (bipolar disorder OR (bipolar AND disorder) OR bipolar disorder*)))) AND ((self-monitor* OR (self-assessment OR self-assess*) OR physiological monitor OR personal monitoring system OR activity tracker OR track* OR screen* OR monitor* OR ecologial momentary assessment))) AND ((wearable* OR (biosensor OR biosensor* OR bio-sensor OR bio-sensor* OR biomedical sensor OR biomedical sensor*))))) OR (((((depress* OR (mood disorder OR (mood AND disorder) OR mood disorder*) OR (affective disorder OR (affective AND disorder) OR affective disorder*) OR (bipolar disorder OR (bipolar AND disorder) OR bipolar disorder*)))) AND ((self-monitor* OR (self-assessment OR self-assess*) OR physiological monitor OR personal monitoring system OR activity tracker OR track* OR screen* OR monitor* OR ecologial momentary assessment))) AND ((prosod* OR (voice analysis OR voice analyses OR voice analys*) OR (speech analysis OR speech analyses OR speech analys*) OR (phonetic analysis OR phonetic analyses OR phonetic analys*))))
