# Supplementary material for: Smartphone-Based Monitoring of Objective and Subjective Data in Affective Disorders: Where Are We and Where Are We Going? Systematic Review
Source: J Med Internet Res. 2017 Jul 24;19(7):e262. doi: 10.2196/jmir.7006 (PMC5547249; doi:10.2196/jmir.7006)
Supplement: Multimedia Appendix 3 [file jmir_v19i7e262_app3.pdf]

Multimedia Appendix 3

Reference list of excluded full text articles

Full text articles were discarded for the following reasons:

A) Reviews

1. aan het Rot, M., Hogenelst, K., & Schoevers, R. A. (2012). Mood disorders in everyday life: a systematic review of experience sampling and ecological momentary assessment studies. *Clinical Psychology Review*, *32*(6), 510–523. https://doi.org/10.1016/j.cpr.2012.05.007

2. Anthes, E. (2016). Mental health: there's an app for that. *Nature*, *532*(7597), 20–23. https://doi.org/10.1038/532020a

3. Armontrout, J., Torous, J., Fisher, M., Drogin, E., & Gutheil, T. (2016). Mobile mental health: navigating new rules and regulations for digital tools. *Current Psychiatry Reports*, *18*(10), 120. https://doi.org/10.1007/s11920-016-0726-x

4. Bakker, D., Kazantzis, N., Rickwood, D., & Rickard, N. (2016). Mental health smartphone apps: review and evidence-based recommendations for future developments. *JMIR Mental Health*, *3*(1), e7. https://doi.org/10.2196/mental.4984

5. Bauer, S., & Moessner, M. (2012). Technology-enhanced monitoring in psychotherapy and e-mental health. *Journal of Mental Health*, *21*(4), 355–363. https://doi.org/10.3109/09638237.2012.667886

6. Ben-Zeev, D., Schueller, S. M., Begale, M., Duffecy, J., Kane, J. M., & Mohr, D. C. (2015). Strategies for mHealth research: lessons from 3 mobile intervention studies. *Administration and Policy in Mental Health*, *42*(2), 157–167. https://doi.org/10.1007/s10488-014-0556-2

7. Berrouiguet, S., Baca-García, E., Brandt, S., Walter, M., & Courtet, P. (2016). Fundamentals for future mobile-health (mHealth): a systematic review of mobile phone and web-based text messaging in mental health. *Journal of Medical Internet Research*, *18*(6), e135. https://doi.org/10.2196/jmir.5066

8. Berry, N., Lobban, F., Emsley, R., & Bucci, S. (2016). Acceptability of interventions delivered online and through mobile phones for people who experience severe mental health problems: a systematic review. *Journal of Medical Internet Research*, *18*(5), e121. https://doi.org/10.2196/jmir.5250

9. Christensen, H., & Petrie, K. (2013). State of the e-mental health field in Australia: where are we now? *Australian & New Zealand Journal of Psychiatry*, *47*(2), 117–120. https://doi.org/10.1177/0004867412471439

10. Christensen, H., Batterham, P., & O'Dea, B. (2014). E-Health interventions for suicide prevention. *International Journal of Environmental Research and Public Health*, *11*(8), 8193–8212. https://doi.org/10.3390/ijerph110808193

11. Clarke, G., & Yarborough, B. J. (2013). Evaluating the promise of health IT to enhance/expand the reach of mental health services. *General Hospital Psychiatry*, *35*(4), 339–344. https://doi.org/10.1016/j.genhosppsych.2013.03.013

12. Clough, B. A., & Casey, L. M. (2015). The smart therapist: a look to the future of smartphones and mHealth technologies in psychotherapy. *Professional Psychology: Research and Practice*, *46*(3), 147–153. https://doi.org/10.1037/pro0000011

13. Coulter, A., Entwistle, V. A., Eccles, A., Ryan, S., Shepperd, S., & Perera, R. (2015). Personalised care planning for adults with chronic or long-term health conditions. *Cochrane Database Syst Rev*, *3*(3), CD010523. https://doi.org/10.1002/14651858.CD010523.pub2

14. Cuijpers, P., & Riper, H. (2014). Internet interventions for depressive disorders: an overview. *Revista de Psicopatología y Psicología Clínica*, *19*(3), 209–216.

15. Cunningham, J. A., Gulliver, A., Farrer, L., Bennett, K., & Carron-Arthur, B. (2014). Internet interventions for mental health and addictions: current findings and future directions. *Current Psychiatry Reports*, *16*(12), 794. https://doi.org/10.1007/s11920-014-0521-5

16. Donker, T., Petrie, K., Proudfoot, J., Clarke, J., Birch, M.-R., & Christensen, H. (2013). Smartphones for smarter delivery of mental health programs: a systematic review. *Journal of Medical Internet Research*, *15*(11), e247. https://doi.org/10.2196/jmir.2791

17. Edelstein, B. A., Woodhead, E. L., Segal, D. L., Heisel, M. J., Bower, E. H., Lowery, A. J., & Stoner, S. A. (2007). Older adult psychological assessment: current instrument status and related considerations. *Clinical Gerontologist*, *31*(3), 1–35. https://doi.org/10.1080/07317110802072108

18. Faurholt-Jepsen, M., Munkholm, K., Frost, M., Bardram, J. E., & Kessing, L. V. (2016). Electronic self-monitoring of mood using IT platforms in adult patients with bipolar disorder: a systematic review of the validity and evidence. *BMC Psychiatry*, *16*(1), 349. https://doi.org/10.1186/s12888-016-0713-0

19. Frueh, B. C. (2015). Solving mental healthcare access problems in the twenty-first century. *Australian Psychologist*, *50*(4), 304–306. https://doi.org/10.1111/ap.12140

20. Gaggioli, A., & Riva, G. (2013). From mobile mental health to mobile wellbeing: opportunities and challenges. In *MMVR* (pp. 141-147).

21. García-Lizana, F., & Muñoz-Mayorga, I. (2010). Telemedicine for depression: a systematic review. *Perspectives in Psychiatric Care*, *46*(2), 119–126. https://doi.org/10.1111/j.1744-6163.2010.00247.x

22. Girard, J. M., & Cohn, J. F. (2015). Automated audiovisual depression analysis. *Current Opinion in Psychology*, *4*, 75–79. https://doi.org/10.1016/j.copsyc.2014.12.010

23. Glenn, T., & Monteith, S. (2014). New measures of mental state and behavior based on data collected from sensors, smartphones, and the internet. *Current Psychiatry Reports*, *16*(12), 202. https://doi.org/10.1007/s11920-014-0523-3

24. Glenn, T., & Monteith, S. (2014). Privacy in the digital world: medical and health data outside of HIPAA protections. *Current Psychiatry Reports*, *16*(11), 657. https://doi.org/10.1007/s11920-014-0494-4

25. Gravenhorst, F., Muaremi, A., Bardram, J., Grünerbl, A., Mayora, O., Wurzer, G.,. . . Tröster, G. (2015). Mobile phones as medical devices in mental disorder treatment: an overview. *Personal and Ubiquitous Computing*, *19*(2), 335–353. https://doi.org/10.1007/s00779-014-0829-5

26. Guest, F. L., Guest, P. C., & Martins-de-Souza, D. (2016). The emergence of point-of-care blood-based biomarker testing for psychiatric disorders: enabling personalized medicine. *Biomarkers in Medicine*, *10*(4), 431–443. https://doi.org/10.2217/bmm-2015-0055

27. Harrison, V., Proudfoot, J., Wee, P. P., Parker, G., Pavlovic, D. H., & Manicavasagar, V. (2011). Mobile mental health: review of the emerging field and proof of concept study. *Journal of Mental Health*, *20*(6), 509–524. https://doi.org/10.3109/09638237.2011.608746

28. Hidalgo-Mazzei, D., Mateu, A., Reinares, M., Matic, A., Vieta, E., & Colom, F. (2015). Internet-based psychological interventions for bipolar disorder: review of the present and insights into the future. *Journal of Affective Disorders*, *188*, 1–13. https://doi.org/10.1016/j.jad.2015.08.005

29. Hill, R. D., Luptak, M. K., Rupper, R. W., Bair, B., Peterson, C., Dailey, N., & Hicken, B. L. (2010). Review of Veterans Health Administration telemedicine interventions. *The American Journal of Managed Care*, *16*(12 Suppl HIT), e302-10.

30. Janney, C. A., Bauer, M. S., & Kilbourne, A. M. (2014). Self-management and bipolar disorder: a clinician's guide to the literature 2011-2014. *Current Psychiatry Reports*, *16*(9), 5. https://doi.org/10.1007/s11920-014-0485-5

31. Keasberry, J., Scott, I. A., Sullivan, C., Staib, A., & Ashby, R. (2017). Going digital: a narrative overview of the clinical and organisational impacts of eHealth technologies in hospital practice. *Australian Health Review.* Advance online publication. https://doi.org/10.1071/AH16233

32. Kempf, M.-C., Huang, C.-H., Savage, R., & Safren, S. A. (2015). Technology-delivered mental health interventions for people living with HIV/AIDS (PLWHA): a review of recent advances. *Current HIV/AIDS Reports*, *12*(4), 472–480. https://doi.org/10.1007/s11904-015-0292-6

33. Khosravi, P., & Ghapanchi, A. H. (2016). Investigating the effectiveness of technologies applied to assist seniors: a systematic literature review. *International Journal of Medical Informatics*, *85*(1), 17–26. https://doi.org/10.1016/j.ijmedinf.2015.05.014

34. Kreuze, E., Jenkins, C., Gregoski, M., York, J., Mueller, M., Lamis, D. A., & Ruggiero, K. J. (2017). Technology-enhanced suicide prevention interventions: a systematic review of the current state of the science. *Journal of Telemedicine and Telecare*, *52*, 1357633X1665792. https://doi.org/10.1177/1357633X16657928

35. Lal, S., & Adair, C. E. (2014). E-mental health: a rapid review of the literature. *Psychiatric Services*, *65*(1), 24–32. https://doi.org/10.1176/appi.ps.201300009

36. Lowe, C. R. (2011). The future: biomarkers, biosensors, neuroinformatics, and e-neuropsychiatry. *International Review of Neurobiology*, *101*, 375–400. https://doi.org/10.1016/B978-0-12-387718-5.00015-8

37. Lukasiewicz, M., Fareng, M., Benyamina, A., Blecha, L., Reynaud, M., & Falissard, B. (2014). Ecological momentary assessment in addiction. *Expert Review of Neurotherapeutics*, *7*(8), 939–950. https://doi.org/10.1586/14737175.7.8.939

38. Malik, A., Goodwin, G. M., & Holmes, E. A. (2012). Contemporary approaches to frequent mood monitoring in bipolar disorder. *Journal of Experimental Psychopathology*, *3*(4), 572–581. https://doi.org/10.5127/jep.014311

39. Marcano Belisario, J. S., Jamsek, J., Huckvale, K., O'Donoghue, J., Morrison, C. P., & Car, J. (2015). Comparison of self-administered survey questionnaire responses collected using mobile apps versus other methods. *The Cochrane Database of Systematic Reviews*. (7), MR000042. https://doi.org/10.1002/14651858.MR000042.pub2

40. Martínez-Pérez, B., La Torre-Díez, I. de, & López-Coronado, M. (2013). Mobile health applications for the most prevalent conditions by the World Health Organization: review and analysis. *Journal of Medical Internet Research*, *15*(6), e120. https://doi.org/10.2196/jmir.2600

41. Marzano, L., Bardill, A., Fields, B., Herd, K., Veale, D., Grey, N., & Moran, P. (2015). The application of mHealth to mental health: opportunities and challenges. *The Lancet Psychiatry*, *2*(10), 942–948. https://doi.org/10.1016/S2215-0366(15)00268-0

42. Matthews, M., Abdullah, S., Gay, G., & Choudhury, T. (2014). Tracking mental well-being: balancing rich sensing and patient needs. *Computer*, *47*(4), 36–43. https://doi.org/10.1109/MC.2014.107

43. McKay, F. H., Cheng, C., Wright, A., Shill, J., Stephens, H., & Uccellini, M. (2017). Evaluating mobile phone applications for health behaviour change: a systematic review. *Journal of Telemedicine and Telecare*, *9*(1), 1357633X1667353. https://doi.org/10.1177/1357633X16673538

44. Mohr, D. C., Burns, M. N., Schueller, S. M., Clarke, G., & Klinkman, M. (2013). Behavioral intervention technologies: evidence review and recommendations for future research in mental health. *General Hospital Psychiatry*, *35*(4), 332–338. https://doi.org/10.1016/j.genhosppsych.2013.03.008

45. Monteith, S., Glenn, T., Geddes, J., & Bauer, M. (2015). Big data are coming to psychiatry: a general introduction. *International Journal of Bipolar Disorders*, *3*(1), 21. https://doi.org/10.1186/s40345-015-0038-9

46. Monteith, S., Glenn, T., Geddes, J., Whybrow, P. C., & Bauer, M. (2016). Big data for bipolar disorder. *International Journal of Bipolar Disorders*, *4*(1), 10. https://doi.org/10.1186/s40345-016-0051-7

47. Morris, M. E., & Aguilera, A. (2012). Mobile, social, and wearable computing and the evolution of psychological practice. *Professional Psychology: Research and Practice*, *43*(6), 622–626. https://doi.org/10.1037/a0029041

48. Naslund, J. A., Marsch, L. A., McHugo, G. J., & Bartels, S. J. (2015). Emerging mHealth and eHealth interventions for serious mental illness: a review of the literature. *Journal of Mental Health*, *24*(5), 321–332. https://doi.org/10.3109/09638237.2015.1019054
49. Nicholas, J., Larsen, M. E., Proudfoot, J., & Christensen, H. (2015). Mobile apps for bipolar disorder: a systematic review of features and content quality. *Journal of Medical Internet Research*, *17*(8), e198. https://doi.org/10.2196/jmir.4581
50. Olff, M. (2015). Mobile mental health: a challenging research agenda. *European Journal of Psychotraumatology*, *6*, 27882. https://doi.org/10.3402/ejpt.v6.27882
51. Overholser, J. (2012). Adapting computerized treatments into traditional psychotherapy for depression. In Wiederhold, B. K., & Riva, G. *Annual Review of Cybertherapy and Telemedicine 2012: Advanced Technologies in the Behavioral, Social and Neurosciences (pp. 32-36).* Doi: 10.3233/978-1-61499-121-2-32
52. Pincus, T. (2016). Electronic multidimensional health assessment questionnaire (eMDHAQ): past, present and future of a proposed single data management system for clinical care, research, quality improvement, and monitoring of long-term outcomes. *Clinical and Experimental Rheumatology*, *34*(5 Suppl 101), S17-S33.
53. Plaza, I., Demarzo, M. M. P., Herrera-Mercadal, P., & García-Campayo, J. (2013). Mindfulness-based mobile applications: literature review and analysis of current features. *JMIR mHealth and uHealth*, *1*(2), e24. https://doi.org/10.2196/mhealth.2733
54. Parikh, S. V., & Huniewicz, P. (2015). E-health: an overview of the uses of the Internet, social media, apps, and websites for mood disorders. *Current Opinion in Psychiatry*, *28*(1), 13–17. https://doi.org/10.1097/YCO.0000000000000123
55. Pentland, A., Lazer, D., Brewer, D., & Heibeck, T. (2009). Using reality mining to improve public health and medicine. *Studies in Health Technology and Informatics*, *149*, 93–102. https://doi.org/10.1378/smbr.4th.93
56. Posadzki, P., Mastellos, N., Ryan, R., Gunn, L. H., Felix, L. M., Pappas, Y.,. . . Car, J. (2016). Automated telephone communication systems for preventive healthcare and management of long-term conditions. *The Cochrane Database of Systematic Reviews*, *12*, CD009921. https://doi.org/10.1002/14651858.CD009921.pub2
57. Price, M., Yuen, E. K., Goetter, E. M., Herbert, J. D., Forman, E. M., Acierno, R., & Ruggiero, K. J. (2014). mHealth: a mechanism to deliver more accessible, more effective mental health care. *Clinical Psychology & Psychotherapy*, *21*(5), 427–436. https://doi.org/10.1002/cpp.1855
58. Proudfoot, J. (2013). The future is in our hands: the role of mobile phones in the prevention and management of mental disorders. *Australian & New Zealand Journal of Psychiatry*, *47*(2), 111–113. https://doi.org/10.1177/0004867412471441
59. Radovic, A., Vona, P. L., Santostefano, A. M., Ciaravino, S., Miller, E., & Stein, B. D. (2016). Smartphone applications for mental health. *Cyberpsychology, Behavior, and Social Networking*, *19*(7), 465–470. https://doi.org/10.1089/cyber.2015.0619

60. Redmond, S. J., Lovell, N. H., Yang, G. Z., Horsch, A., Lukowicz, P., Murrugarra, L., & Marschollek, M. (2014). What does big data mean for wearable sensor systems?: Contribution of the IMIA wearable sensors in healthcare WG. *Yearbook of Medical Informatics*, *9*(1), 135. https://doi.org/10.4172/2157-7420.S1.004

61. Reynolds, J., Griffiths, K., & Christensen, H. (2011). Anxiety and depression - online resources and management tools. *Australian Family Physician*, *40*(6), 382–386.

62. Riva, G., Baños, R. M., Botella, C., Gaggioli, A., & Wiederhold, B. K. (2011). Personal health systems for mental health: the European projects. In *MMVR* (pp. 496-502).

63. Sandstrom, G. M., Lathia, N., Mascolo, C., & Rentfrow, P. J. (2016). Opportunities for smartphones in clinical care: the future of mobile mood monitoring. *The Journal of Clinical Psychiatry*, *77*(2), e135-7. https://doi.org/10.4088/JCP.15com10054

64. Scott, K., & Lewis, C. C. (2015). Using measurement-based care to enhance any treatment. *Cognitive and Behavioral Practice*, *22*(1), 49–59. https://doi.org/10.1016/j.cbpra.2014.01.010

65. Seko, Y., Kidd, S., Wiljer, D., & McKenzie, K. (2014). Youth mental health interventions via mobile phones: a scoping review. *Cyberpsychology, Behavior, and Social Networking*, *17*(9), 591–602. https://doi.org/10.1089/cyber.2014.0078

66. Shapiro, J. R., & Bauer, S. Use of short message service (SMS)-based interventions to enhance low intensity CBT, pp. 281–286. https://doi.org/10.1093/med:psych/9780199590117.003.0028

67. Shen, N., Levitan, M.-J., Johnson, A., Bender, J. L., Hamilton-Page, M., Jadad, A. R., & Wiljer, D. (2015). Finding a depression app: a review and content analysis of the depression app marketplace. *JMIR mHealth and uHealth*, *3*(1), e16. https://doi.org/10.2196/mhealth.3713

68. Shore, J. H. (2015). The technological transformation of psychiatric care: telepsychiatry comes of age. *International Review of Psychiatry*, *27*(6), 467–468. https://doi.org/10.3109/09540261.2015.1120008

69. Vahabzadeh, A., Sahin, N., & Kalali, A. (2016). Digital suicide prevention: can technology become a game-changer? *Innovations in Clinical Neuroscience*, *13*(5-6), 16–20.

70. Vallury, K. D., Jones, M., & Oosterbroek, C. (2015). Computerized cognitive behavior therapy for anxiety and depression in rural areas: a systematic review. *Journal of Medical Internet Research*, *17*(6), e139. https://doi.org/10.2196/jmir.4145

71. Versluis, A., Verkuil, B., Spinhoven, P., van der Ploeg, Melanie M, & Brosschot, J. F. (2016). Changing mental health and positive psychological well-being using ecological momentary interventions: a systematic review and meta-analysis. *Journal of Medical Internet Research*, *18*(6), e152. https://doi.org/10.2196/jmir.5642

72. Walsh, S., Golden, E., & Priebe, S. (2016). Systematic review of patients' participation in and experiences of technology-based monitoring of mental health symptoms in the community. *BMJ Open*, *6*(6), e008362. https://doi.org/10.1136/bmjopen-2015-008362

73. Wenze, S. J., & Miller, I. W. (2010). Use of ecological momentary assessment in mood disorders research. *Clinical Psychology Review*, *30*(6), 794–804. https://doi.org/10.1016/j.cpr.2010.06.007

74. Wichers, M., Simons, C. J. P., Kramer, I. M. A., Hartmann, J. A., Lothmann, C., Myin-Germeys, I.,. . . van Os, J. (2011). Momentary assessment technology as a tool to help patients with depression help themselves. *Acta Psychiatrica Scandinavica*, *124*(4), 262–272. https://doi.org/10.1111/j.1600-0447.2011.01749.x

75. Williams Jr, J. W., & Manning, J. S. (2008). Collaborative mental health and primary care for bipolar disorder. *Journal of Psychiatric Practice*, *14*, 55–64.

B) Study protocols OR only study intend mentioned

1. Ask, P., Ekstrand, K., Hult, P., Linden, M., & Pettersson, N.-E. (2012). NovaMedTech - a regional program for supporting new medical technologies in personalized health care. *Studies in Health Technology and Informatics*, *177*, 71–75.

2. Bardram, J. E., Frost, M., Szántó, K., & Marcu, G. The MONARCA self-assessment system: a persuasive personal monitoring system for bipolar patients: Proceedings of the 2nd ACM SIGHIT International Health Informatics Symposium (pp. 21-30). ACM. 2012, 21. https://doi.org/10.1145/2110363.2110370

3. Broglia, E., Millings, A., & Barkham, M. (2017). Comparing counselling alone versus counselling supplemented with guided use of a well-being app for university students experiencing anxiety or depression (CASELOAD): protocol for a feasibility trial. *Pilot and Feasibility Studies*, *3*, 3. https://doi.org/10.1186/s40814-016-0119-2

4. Faurholt-Jepsen, M., Vinberg, M., Frost, M., Christensen, E. M., Bardram, J., & Kessing, L. V. (2014). Daily electronic monitoring of subjective and objective measures of illness activity in bipolar disorder using smartphones– the MONARCA II trial protocol: a randomized controlled single-blind parallel-group trial. *BMC Psychiatry*, *14*(1), 421. https://doi.org/10.1186/s12888-014-0309-5

5. Faurholt-Jepsen, M., Vinberg, M., Christensen, E. M., Frost, M., Bardram, J., & Kessing, L. V. (2013). Daily electronic self-monitoring of subjective and objective symptoms in bipolar disorder—the MONARCA trial protocol (MONitoring, treAtment and pRediCtion of bipolAr disorder episodes): a randomised controlled single-blind trial. *BMJ Open*, *3*(7), e003353. https://doi.org/10.1136/bmjopen-2013-003353

6. Fletcher, R. R., Poh, M. Z., & Eydgahi, H. (2010, August). Wearable sensors: opportunities and challenges for low-cost health care. In *Engineering in Medicine and Biology Society (EMBC), 2010 Annual International Conference of the IEEE* (pp. 1763-1766). IEEE.

7. Gluhak, A., Presser, M., Zhu, L., Esfandiyari, S., & Kupschick, S. (2007, October). Towards mood based mobile services and applications. In *European Conference on Smart Sensing and Context* (pp. 159-174). Springer Berlin Heidelberg.

8. Hidalgo-Mazzei, D., Mateu, A., Reinares, M., Undurraga, J., Bonnín, C. d. M., Sánchez-Moreno, J.,. . . Colom, F. (2015). Self-monitoring and psychoeducation in bipolar patients with a smart-phone application (SIMPLe) project: design, development and studies protocols. *BMC Psychiatry*, *15*(1), 1337. https://doi.org/10.1186/s12888-015-0437-6

9. Kordy, H., Backenstrass, M., Hüsing, J., Wolf, M., Aulich, K., Bürgy, M.,. . . Vedder, H. (2013). Supportive monitoring and disease management through the internet: an internet-delivered intervention strategy for recurrent depression. *Contemporary Clinical Trials*, *36*(2), 327–337. https://doi.org/10.1016/j.cct.2013.08.005

10. Lam, K.-Y., Wang, J., Ng, J. K.-Y., Han, S., Zheng, L., Kam, C. H. C., & Zhu, C. J. (2015). SmartMood: toward pervasive mood tracking and analysis for manic episode detection. *IEEE Transactions on Human-Machine Systems*, *45*(1), 126–131. https://doi.org/10.1109/THMS.2014.2360469

11. Lewis, C. C., Scott, K., Marti, C. N., Marriott, B. R., Kroenke, K., Putz, J. W.,. . . Rutkowski, D. (2015). Implementing measurement-based care (iMBC) for depression in community mental health: a dynamic cluster randomized trial study protocol. *Implementation Science*, *10*(1), 288. https://doi.org/10.1186/s13012-015-0313-2

12. Liapi, M., Linaraki, D., & Voradaki, G. (2012). Sensponsive architecture as a tool to stimulate the senses and alleviate the psychological disorders of an individual. *Cognitive Processing*, *13 Suppl 1*, S233-7. https://doi.org/10.1007/s10339-012-0454-z

13. Markowetz, A., Błaszkiewicz, K., Montag, C., Switala, C., & Schlaepfer, T. E. (2014). Psycho-Informatics: big Data shaping modern psychometrics. *Medical Hypotheses*, *82*(4), 405–411. https://doi.org/10.1016/j.mehy.2013.11.030

14. Massey, T., Marfia, G., Potkonjak, M., & Sarrafzadeh, M. (2010). Experimental analysis of a mobile health system for mood disorders. *IEEE transactions on Information Technology in Biomedicine*, *14*(2), 241–247. https://doi.org/10.1109/TITB.2009.2034738

15. Matthews, M., Abdullah, S., Murnane, E., Voida, S., Choudhury, T., Gay, G., & Frank, E. (2016). Development and evaluation of a smartphone-based measure of social rhythms for bipolar disorder. *Assessment*, *23*(4), 472–483. https://doi.org/10.1177/1073191116656794

16. Paradiso, R., Bianchi, A. M., Lau, K., & Scilingo, E. P. (2010, August). Psyche: personalised monitoring systems for care in mental health. In *Engineering in Medicine and Biology Society (EMBC), 2010 annual international conference of the IEEE* (pp. 3602-3605). IEEE.

17. Paradiso, R., Faetti, T., & Werner, S. (2011, August). Wearable monitoring systems for psychological and physiological state assessment in a naturalistic environment. In *Engineering in Medicine and Biology Society, EMBC, 2011 Annual International Conference of the IEEE* (pp. 2250-2253). IEEE.

18. Prociow, P., & Crowe, J. (2009, July). Sensors enhancing self-monitoring for people with bipolar disorder. In *Proc. 5th UKRI PG Conf in Biomedical Engineering and Medical Physics, Oxford* (pp. 37-38).

19. Puiatti, A., Mudda, S., Giordano, S., & Mayora, O. Smartphone-centred wearable sensors network for monitoring patients with bipolar disorder, pp. 3644–3647. https://doi.org/10.1109/IEMBS.2011.6090613

20. Sander, L., Paganini, S., Lin, J., Schlicker, S., Ebert, D. D., Buntrock, C., & Baumeister, H. (2017). Effectiveness and cost-effectiveness of a guided Internet- and mobile-based intervention for the indicated prevention of major depression in patients with chronic back pain—study protocol of the PROD-BP multicenter pragmatic RCT. *BMC Psychiatry*, *17*(1), 36. https://doi.org/10.1186/s12888-017-1193-6

21. Valenza, G., Lanata, A., Paradiso, R., & Scilingo, E. P. (2014). Advanced technology meets mental health: how smartphones, textile electronics, and signal processing can serve mental health monitoring, diagnosis, and treatment. *IEEE Pulse*, *5*(3), 56–59. https://doi.org/10.1109/MPUL.2014.2309582

22. van Ballegooijen, W., Ruwaard, J., Karyotaki, E., Ebert, D. D., Smit, J. H., & Riper, H. (2016). Reactivity to smartphone-based ecological momentary assessment of depressive symptoms (MoodMonitor): protocol of a randomised controlled trial. *BMC Psychiatry*, *16*(1), 457. https://doi.org/10.1186/s12888-016-1065-5

23. van de Ven, P., Henriques, M. R., Hoogendoorn, M., Klein, M., McGovern, E., Nelson, J.,. . . Tousset, E. (2012). A mobile system for treatment of depression. *Computing Paradigms for Mental Health*, 47.

24. Warmerdam, L., Riper, H., Klein, M., van den Ven, P., Rocha, A., Ricardo Henriques, M.,. . . Cuijpers, P. (2012). Innovative ICT solutions to improve treatment outcomes for depression: the ICT4Depression project. *Studies in Health Technology and Informatics*, *181*, 339–343.

25. Wilansky, P., Eklund, J. M., Milner, T., Kreindler, D., Cheung, A., Kovacs, T.,. . . Mills, R. S. (2016). Cognitive behavior therapy for anxious and depressed youth: improving homework adherence through mobile technology. *JMIR Research Protocols*, *5*(4), e209. https://doi.org/10.2196/resprot.5841

26. Wilhelm, F. H., Roth, W. T., & Sackner, M. A. (2003). The LifeShirt: an advanced system for ambulatory measurement of respiratory and cardiac function. *Behavior Modification*, *27*(5), 671–691. https://doi.org/10.1177/0145445503256321

27. Wolters, M. K., Martinez-Miranda, J., Hastie, H. F., & Matheson, C. (2012). Managing data in Help4mood. *Computing Paradigms for Mental Health*, 17.

C) No participants with diagnosed affective disorder

1. Ainsworth, J., Palmier-Claus, J. E., Machin, M., Barrowclough, C., Dunn, G., Rogers, A.,. . . Lewis, S. (2013). A comparison of two delivery modalities of a mobile phone-based assessment for serious mental illness: native smartphone application vs text-

messaging only implementations. *Journal of Medical Internet Research*, *15*(4), e60. https://doi.org/10.2196/jmir.2328

2. Alam, M. G. R., Abedin, S. F., Al Ameen, M., & Hong, C. S. (2016). Web of objects based ambient assisted living framework for emergency psychiatric state prediction. *Sensors*, *16*(9). https://doi.org/10.3390/s16091431
3. Alam, M. G. R., Cho, E. J., Huh, E. N., & Hong, C. S. (2014). *Cloud based mental state monitoring system for suicide risk reconnaissance using wearable bio-sensors.: In Proceedings of the 8th International Conference on Ubiquitous Information Management and Communication (p. 56)*: ACM.
4. Andone, I., Błaszkiewicz, K., Eibes, M., Trendafilov, B., Montag, C., & Markowetz, A. (2016). Menthal-Running a Science Project as a Start-Up. In *Computing in Mental Health, Workshop at CHI 2016*.
5. Anguera, J. A., Jordan, J. T., Castaneda, D., Gazzaley, A., & Areán, P. A. (2016). Conducting a fully mobile and randomised clinical trial for depression: access, engagement and expense. *BMJ Innovations*, *2*(1), 14–21. https://doi.org/10.1136/bmjinnov-2015-000098
6. Arean, P. A., Hallgren, K. A., Jordan, J. T., Gazzaley, A., Atkins, D. C., Heagerty, P. J., & Anguera, J. A. (2016). The use and effectiveness of mobile apps for depression: results from a fully remote clinical trial. *Journal of Medical Internet Research*, *18*(12), e330. https://doi.org/10.2196/jmir.6482
7. Asselbergs, J., Ruwaard, J., Ejdys, M., Schrader, N., Sijbrandij, M., & Riper, H. (2016). Mobile phone-based unobtrusive ecological momentary assessment of day-to-day mood: an explorative study. *Journal of Medical Internet Research*, *18*(3), e72. https://doi.org/10.2196/jmir.5505
8. Bauer, A. M., Rue, T., Keppel, G. A., Cole, A. M., Baldwin, L.-M., & Katon, W. (2014). Use of mobile health (mHealth) tools by primary care patients in the WWAMI region practice and research network (WPRN). *The Journal of the American Board of Family Medicine*, *27*(6), 780–788. https://doi.org/10.3122/jabfm.2014.06.140108
9. Ben-Zeev, D., Scherer, E. A., Wang, R., Xie, H., & Campbell, A. T. (2015). Next-generation psychiatric assessment: Using smartphone sensors to monitor behavior and mental health. *Psychiatric Rehabilitation Journal*, *38*(3), 218–226. https://doi.org/10.1037/prj0000130
10. BinDhim, N. F., Alanazi, E. M., Aljadhey, H., Basyouni, M. H., Kowalski, S. R., Pont, L. G.,. . . Alhawassi, T. M. (2016). Does a mobile phone depression-screening app motivate mobile phone users with high depressive symptoms to seek a health care professional’s help? *Journal of Medical Internet Research*, *18*(6), e156. https://doi.org/10.2196/jmir.5726
11. Birney, A. J., Gunn, R., Russell, J. K., & Ary, D. V. (2016). MoodHacker mobile web app with email for adults to self-manage mild-to-moderate depression: randomized controlled trial. *JMIR mHealth and uHealth*, *4*(1), e8. https://doi.org/10.2196/mhealth.4231
12. Botella, C., Etchemendy, E., Castilla, D., Baños, R. M., García-Palacios, A., Quero, S.,. . . Lozano, J. A. (2009). An e-Health system for the elderly (Butler Project): a pilot

study on acceptance and satisfaction. *CyberPsychology & Behavior*, *12*(3), 255–262. https://doi.org/10.1089/cpb.2008.0325

13. Braun, S., Annovazzi, C., Botella, C., Bridler, R., Camussi, E., Delfino, J. P.,. . . Stassen, H. H. (2017). Assessing chronic stress, coping skills, and mood disorders through speech analysis: a self-assessment 'Voice App' for laptops, tablets, and smartphones. *Psychopathology*, *49*(6), 406–419. https://doi.org/10.1159/000450959

14. Braun, S., Botella, C., Bridler, R., Chmetz, F., Delfino, J. P., Herzig, D.,. . . Stassen, H. H. (2014). Affective state and voice: cross-cultural assessment of speaking behavior and voice sound characteristics - a normative multicenter study of 577 + 36 healthy subjects. *Psychopathology*, *47*(5), 327–340. https://doi.org/10.1159/000363247

15. Burns, M. N., Montague, E., & Mohr, D. C. (2013). Initial design of culturally informed behavioral intervention technologies: developing an mHealth intervention for young sexual minority men with generalized anxiety disorder and major depression. *Journal of Medical Internet Research*, *15*(12), e271. https://doi.org/10.2196/jmir.2826

16. Bush, N. E., Ouellette, G., & Kinn, J. (2014). Utility of the T2 Mood Tracker Mobile Application among army warrior transition unit service members. *Military Medicine*, *179*(12), 1453–1457. https://doi.org/10.7205/MILMED-D-14-00271

17. Chang, K. H., Fisher, D., Canny, J., & Hartmann, B. (Eds.). (2011, November). *How's my mood and stress?: an efficient speech analysis library for unobtrusive monitoring on mobile phones. In Proceedings of the 6th International Conference on Body Area Networks (pp. 71-77).*: ICST (Institute for Computer Sciences, Social-Informatics and Telecommunications Engineering).

18. Chiu, C.-J., Hu, Y.-H., Lin, D.-C., Chang, F.-Y., Chang, C.-S., & Lai, C.-F. (2016). The attitudes, impact, and learning needs of older adults using apps on touchscreen mobile devices: Results from a pilot study. *Computers in Human Behavior*, *63*, 189–197. https://doi.org/10.1016/j.chb.2016.05.020

19. Chouvarda, I., Philip, N. Y., Natsiavas, P., Kilintzis, V., Sobnath, D., Kayyali, R.,. . . Maglaveras, N. (2014, August). *WELCOME—innovative integrated care platform using wearable sensing and smart cloud computing for COPD patients with comorbidities. In Engineering in Medicine and Biology Society (EMBC), 2014 36th Annual International Conference of the IEEE (pp. 3180-3183)*: IEEE.

20. Christensen, M. A., Bettencourt, L., Kaye, L., Moturu, S. T., Nguyen, K. T., Olgin, J. E.,. . . Romigi, A. (2016). Direct measurements of smartphone screen-time: relationships with demographics and sleep. *PLoS ONE*, *11*(11), e0165331. https://doi.org/10.1371/journal.pone.0165331

21. Clarke, J., Proudfoot, J., & Ma, H. (2016). Mobile phone and web-based cognitive behavior therapy for depressive symptoms and mental health comorbidities in people living with diabetes: results of a feasibility study. *JMIR Mental Health*, *3*(2), e23. https://doi.org/10.2196/mental.5131

22. Clarke, J., Proudfoot, J., Whitton, A., Birch, M.-R., Boyd, M., Parker, G.,. . . Fogarty, A. (2016). Therapeutic alliance with a fully automated mobile phone and web-based

intervention: secondary analysis of a randomized controlled trial. *JMIR Mental Health*, *3*(1), e10. https://doi.org/10.2196/mental.4656

23. Clarke, J., Vatiliotis, V., Verge, C. F., Holmes-Walker, J., Campbell, L. V., Wilhelm, K., & Proudfoot, J. (2015). A mobile phone and web-based intervention for improving mental well-being in young people with type 1 diabetes: design of a randomized controlled trial. *JMIR Research Protocols*, *4*(2), e50. https://doi.org/10.2196/resprot.4032

24. Clasen, P. C., Fisher, A. J., Beevers, C. G., & Lui, S. (2015). Mood-reactive self-esteem and depression vulnerability: person-specific symptom dynamics via smart phone assessment. *PLoS ONE*, *10*(7), e0129774. https://doi.org/10.1371/journal.pone.0129774

25. Cook, B. L., Progovac, A. M., Chen, P., Mullin, B., Hou, S., & Baca-Garcia, E. (2016). Novel use of Natural Language Processing (NLP) to predict suicidal ideation and psychiatric symptoms in a text-based mental health intervention in Madrid. *Computational and Mathematical Methods in Medicine*, *2016*(9859), 1–8. https://doi.org/10.1155/2016/8708434

26. de Cerio, D. P.-D., Boque, S. R., Rosell-Ferrer, J., Ramoscastro, J., Valenzuela, J. L., & Colome, J. M. (2013). The help4mood wearable sensor network for inconspicuous activity measurement. *IEEE Wireless Communications*, *20*(4), 50–56. https://doi.org/10.1109/MWC.2013.6590050

27. de Luca, R., Bramanti, A., de Cola, M. C., Trifiletti, A., Tomasello, P., Torrisi, M.,. . . Calabro, R. S. (2016). Tele-health-care in the elderly living in nursing home: the first Sicilian multimodal approach. *Aging Clinical and Experimental Research*, *28*(4), 753–759. https://doi.org/10.1007/s40520-015-0463-8

28. Deady, M., Mills, K. L., Teesson, M., & Kay-Lambkin, F. (2016). An online intervention for co-occurring depression and problematic alcohol use in young people: primary outcomes from a randomized controlled trial. *Journal of Medical Internet Research*, *18*(3), e71. https://doi.org/10.2196/jmir.5178

29. Dutta, A., Kumar, R., Malhotra, S., Chugh, S., Banerjee, A., & Dutta, A. (2013). A low-cost point-of-care testing system for psychomotor symptoms of depression affecting standing balance: a preliminary study in India. *Depression Research and Treatment*, *2013*(3), 1–8. https://doi.org/10.1155/2013/640861

30. Fafoutis, X., Tsimbalo, E., Mellios, E., Hilton, G., Piechocki, R., & Craddock, I. (2016). A residential maintenance-free long-term activity monitoring system for healthcare applications. *EURASIP Journal on Wireless Communications and Networking*, *1*, 31. https://doi.org/10.1186/s13638-016-0534-3

31. Fanning, J., Mackenzie, M., Roberts, S., Crato, I., Ehlers, D., & McAuley, E. (2016). Physical activity, mind wandering, affect, and sleep: an ecological momentary assessment. *JMIR mHealth and uHealth*, *4*(3), e104. https://doi.org/10.2196/mhealth.5855

32. Farrer, L., Christensen, H., Griffiths, K. M., & Mackinnon, A. (2012). Web-based cognitive behavior therapy for depression with and without telephone tracking in a

national helpline: secondary outcomes from a randomized controlled trial. *Journal of Medical Internet Research*, *14*(3), e68. https://doi.org/10.2196/jmir.1859

33. Fernandez, K. C., Johnson, M. R., & Rodebaugh, T. L. (2013). TelEMA: a low-cost and user-friendly telephone assessment platform. *Behavior Research Methods*, *45*(4), 1279–1291.

34. Fletcher, R. R., Dobson, K., Goodwin, M. S., Eydgahi, H., Wilder-Smith, O., Fernholz, D.,. . . Picard, R. W. (2010). iCalm: wearable sensor and network architecture for wirelessly communicating and logging autonomic activity. *IEEE transactions on Information Technology in Biomedicine*, *14*(2), 215–223. https://doi.org/10.1109/TITB.2009.2038692

35. Froehlich, J., Chen, M. Y., Consolvo, S., Harrison, B., & Landay, J. A. (2007, June). MyExperience: a system for in situ tracing and capturing of user feedback on mobile phones. In *Proceedings of the 5th international conference on Mobile systems, applications and services* (pp. 57-70). ACM.

36. Funane, T. Wearable near-infrared spectroscopy neuroimaging and its applications, pp. 4025–4028. https://doi.org/10.1109/EMBC.2015.7319277

37. Fuster-Garcia, E., Bresó, A., Martínez-Miranda, J., Rosell-Ferrer, J., Matheson, C., & García-Gómez, J. M. (2015). Fusing actigraphy signals for outpatient monitoring. *Information Fusion*, *23*, 69–80. https://doi.org/10.1016/j.inffus.2014.08.003

38. Gaggioli, A., Cipresso, P., Serino, S., Pioggia, G., Tartarisco, G., Baldus, G., Corda, D., Riva, G.. (2012) . An open source mobile platform for psychophysiological self tracking. In Westwood, J. D., Westwood, S.W., Felländer-Tsai, L., Haluck, R.S., Robb, R.A., Senger, S., Vosburgh, K.G. *Medicine Meets Virtual Reality 19: NextMed*, *(pp.* 136-138). Doi: 10.3233/978-1-61499-022-2-136

39. Gaggioli, A., Pioggia, G., Tartarisco, G., Baldus, G., Corda, D., Cipresso, P., & Riva, G. (2013). A mobile data collection platform for mental health research. *Personal and Ubiquitous Computing*, *17*(2), 241–251. https://doi.org/10.1007/s00779-013-0650-6

40. Giosan, C., Mogoase, C., Cobeanu, O., Szentagotai Tatar, A., Muresan, V., & Boian, R. (2016). Using a smartphone app to reduce cognitive vulnerability and mild depressive symptoms: study protocol of an exploratory randomized controlled trial. *Trials*, *17*(1), 609. https://doi.org/10.1186/s13063-016-1740-3

41. Guiry, J. J., Warmerdam, L., van der Hilst, P., Riper, H., van de Ven, P., & Nelson, J. (2012). The Role of Smartphones as an Assistive Aid in Mental Health. *Computing Paradigms for Mental Health*, 105.

42. Harwood, J., Dooley, J. J., Scott, A. J., & Joiner, R. (2014). Constantly connected – the effects of smart-devices on mental health. *Computers in Human Behavior*, *34*, 267–272. https://doi.org/10.1016/j.chb.2014.02.006

43. Huang, Y.-P., Huang, C.-Y., & Liu, S.-I. (2014). Hybrid intelligent methods for arrhythmia detection and geriatric depression diagnosis. *Applied Soft Computing*, *14*, 38–46. https://doi.org/10.1016/j.asoc.2013.09.021

44. Imamura, K., Kawakami, N., Furukawa, T. A., Matsuyama, Y., Shimazu, A., Umanodan, R.,. . . Jiménez-Murcia, S. (2014). Effects of an internet-based cognitive

behavioral therapy (iCBT) program in manga format on improving subthreshold depressive symptoms among healthy workers: a randomized controlled trial. *PLoS ONE*, *9*(5), e97167. https://doi.org/10.1371/journal.pone.0097167

45. Ivorra, A., Daniels, C., & Rubinsky, B. (2008). Minimally obtrusive wearable device for continuous interactive cognitive and neurological assessment. *Physiological Measurement*, *29*(5), 543–554. https://doi.org/10.1088/0967-3334/29/5/002

46. Janevic, M. R., Aruquipa Yujra, A. C., Marinec, N., Aguilar, J., Aikens, J. E., Tarrazona, R., & Piette, J. D. (2016). Feasibility of an interactive voice response system for monitoring depressive symptoms in a lower-middle income Latin American country. *International Journal of Mental Health Systems*, *10*(1), 111. https://doi.org/10.1186/s13033-016-0093-3

47. Juengst, S. B., Graham, K. M., Pulantara, I. W., McCue, M., Whyte, E. M., Dicianno, B. E.,. . . Wagner, A. K. (2015). Pilot feasibility of an mHealth system for conducting ecological momentary assessment of mood-related symptoms following traumatic brain injury. *Brain Injury*, *29*(11), 1351–1361. https://doi.org/10.3109/02699052.2015.1045031

48. Kappeler-Setz, C., Gravenhorst, F., Schumm, J., Arnrich, B., & Tröster, G. (2013). Towards long term monitoring of electrodermal activity in daily life. *Personal and Ubiquitous Computing*, *17*(2), 261–271.

49. Kauer, S. D., Reid, S. C., Crooke, A. H. D., Khor, A., Hearps, S. J. C., Jorm, A. F.,. . . Patton, G. (2012). Self-monitoring using mobile phones in the early stages of adolescent depression: randomized controlled trial. *Journal of Medical Internet Research*, *14*(3), e67. https://doi.org/10.2196/jmir.1858

50. Kayyali, R., Savickas, V., Spruit, M. A., Kaimakamis, E., Siva, R., Costello, R. W.,. . . Nabhani-Gebara, S. (2016). Qualitative investigation into a wearable system for chronic obstructive pulmonary disease: the stakeholders' perspective. *BMJ Open*, *6*(8), e011657. https://doi.org/10.1136/bmjopen-2016-011657

51. Kelders, S. M., Bohlmeijer, E. T., Pots, W. T., & van Gemert-Pijnen, J. E. (2015). Comparing human and automated support for depression: fractional factorial randomized controlled trial. *Behaviour Research and Therapy*, *72*, 72–80. https://doi.org/10.1016/j.brat.2015.06.014

52. Kenny, R., Dooley, B., & Fitzgerald, A. (2016). Ecological momentary assessment of adolescent problems, coping efficacy, and mood states using a mobile phone app: an exploratory study. *JMIR Mental Health*, *3*(4), e51. https://doi.org/10.2196/mental.6361

53. Kim, J., Lim, S., Min, Y. H., Shin, Y.-W., Lee, B., Sohn, G.,. . . Lee, J. W. (2016). Depression screening using daily mental-health ratings from a smartphone application for breast cancer patients. *Journal of Medical Internet Research*, *18*(8), e216. https://doi.org/10.2196/jmir.5598

54. Kim, J., Nakamura, T., Kikuchi, H., Sasaki, T., Yamamoto, Y., & Mazza, M. (2013). Co-variation of depressive mood and locomotor dynamics evaluated by ecological momentary assessment in healthy humans. *PLoS ONE*, *8*(9), e74979. https://doi.org/10.1371/journal.pone.0074979

55. Lane, N. D., Mohammod, M., Lin, M., Yang, X., Lu, H., Ali, S., ... & Campbell, A. (2011, May). Bewell: A smartphone application to monitor, model and promote wellbeing. In *5th international ICST conference on pervasive computing technologies for healthcare* (pp. 23-26).

56. Lappalainen, P., Kaipainen, K., Lappalainen, R., Hoffrén, H., Myllymäki, T., Kinnunen, M.-L.,. . . Eysenbach, G. (2012). Feasibility of a personal health technology-based psychological intervention for men with stress and mood problems: randomized controlled pilot trial. *JMIR Research Protocols*, *2*(1), e1. https://doi.org/10.2196/resprot.2389

57. Lattie, E. G., Schueller, S. M., Sargent, E., Stiles-Shields, C., Tomasino, K. N., Corden, M. E.,. . . Mohr, D. C. (2016). Uptake and usage of IntelliCare: a publicly available suite of mental health and well-being apps. *Internet Interventions*, *4*, 152–158. https://doi.org/10.1016/j.invent.2016.06.003

58. Lee, M. D., Kang, X., & Hanrahan, N. (2014). Addressing cultural contexts in the management of stress via narrative and mobile technology. *Studies in Health Technology and Informatics*, *199*, 173–177.

59. LiKamWa, R., Liu, Y., Lane, N. D., & Zhong, L. (2013, June). Moodscope: Building a mood sensor from smartphone usage patterns. In *Proceeding of the 11th annual international conference on Mobile systems, applications, and services* (pp. 389-402). ACM.

60. Ma, Y., Xu, B., Bai, Y., Sun, G., & Zhu, R. (2012, May). Daily mood assessment based on mobile phone sensing. In *Wearable and implantable body sensor networks (BSN), 2012 ninth international conference on* (pp. 142-147). IEEE.

61. Meinlschmidt, G., Lee, J.-H., Stalujanis, E., Belardi, A., Oh, M., Jung, E. K.,. . . Tegethoff, M. (2016). Smartphone-based psychotherapeutic micro-interventions to improve mood in a real-world setting. *Frontiers in Psychology*, *7*, 1112. https://doi.org/10.3389/fpsyg.2016.01112

62. Merilahti, J., Pärkkä, J., Antila, K., Paavilainen, P., Mattila, E., Malm, E.-J.,. . . Korhonen, I. (2009). Compliance and technical feasibility of long-term health monitoring with wearable and ambient technologies. *Journal of Telemedicine and Telecare*, *15*(6), 302–309. https://doi.org/10.1258/jtt.2009.081106

63. Miner, A. S., Milstein, A., Schueller, S., Hegde, R., Mangurian, C., & Linos, E. (2016). Smartphone-based conversational agents and responses to questions about mental health, interpersonal violence, and physical health. *JAMA Internal Medicine*, *176*(5), 619–625. https://doi.org/10.1001/jamainternmed.2016.0400

64. Mochari-Greenberger, H., Vue, L., Luka, A., Peters, A., & Pande, R. L. (2016). A tele-behavioral health intervention to reduce depression, anxiety, and stress and improve diabetes self-management. *Telemedicine and e-Health*, *22*(8), 624–630. https://doi.org/10.1089/tmj.2015.0231

65. Mohiuddin, S. G., Brailsford, S. C., James, C. J., Amor, J. D., Blum, J. M., Crowe, J. A.,. . . Prociow, P. A. (2013). A multi-state model to improve the design of an automated system to monitor the activity patterns of patients with bipolar disorder.

Journal of the Operational Research Society, *64*(3), 372–383. https://doi.org/10.1057/jors.2012.57

66. Mohr, D. C., Tomasino, K. N., Lattie, E. G., Palac, H. L., Kwasny, M. J., Weingardt, K.,. . . Schueller, S. M. (2017). IntelliCare: an eclectic, skills-based app suite for the treatment of depression and anxiety. *Journal of Medical Internet Research*, *19*(1), e10. https://doi.org/10.2196/jmir.6645

67. Moore, R. C., Depp, C. A., Wetherell, J. L., & Lenze, E. J. (2016). Ecological momentary assessment versus standard assessment instruments for measuring mindfulness, depressed mood, and anxiety among older adults. *Journal of Psychiatric Research*, *75*, 116–123. https://doi.org/10.1016/j.jpsychires.2016.01.011

68. Morris, M. E., Kathawala, Q., Leen, T. K., Gorenstein, E. E., Guilak, F., Labhard, M., & Deleeuw, W. (2010). Mobile therapy: case study evaluations of a cell phone application for emotional self-awareness. *Journal of Medical Internet Research*, *12*(2), e10. https://doi.org/10.2196/jmir.1371

69. Ory, M. G., Ahn, S., Jiang, L., Lorig, K., Ritter, P., Laurent, D. D.,. . . Smith, M. L. (2013). National Study of Chronic Disease Self-Management. *Journal of Aging and Health*, *25*(7), 1258–1274. https://doi.org/10.1177/0898264313502531

70. Price, M., Sawyer, T., Harris, M., & Skalka, C. (2016). Usability evaluation of a mobile monitoring system to assess symptoms after a traumatic injury: a mixed-methods study. *JMIR Mental Health*, *3*(1), e3. https://doi.org/10.2196/mental.5023

71. Prociow, P. A., & Crowe, J. A. (2010). Towards personalised ambient monitoring of mental health via mobile technologies. *Technology and Health Care*, *18*(4-5), 275–284. https://doi.org/10.3233/THC-2010-0590

72. Prociow, P. A., & Crowe, J. A. (2010, August). Development of mobile psychiatry for bipolar disorder patients. In *Engineering in Medicine and Biology Society (EMBC), 2010 Annual International Conference of the IEEE* (pp. 5484-5487). IEEE.

73. Proudfoot, J., Clarke, J., Birch, M.-R., Whitton, A. E., Parker, G., Manicavasagar, V.,. . . Hadzi-Pavlovic, D. (2013). Impact of a mobile phone and web program on symptom and functional outcomes for people with mild-to-moderate depression, anxiety and stress: a randomised controlled trial. *BMC Psychiatry*, *13*(1), 593. https://doi.org/10.1186/1471-244X-13-312

74. Rabbi, M., Ali, S., Choudhury, T., & Berke, E. (2011, September). Passive and in-situ assessment of mental and physical well-being using mobile sensors. In *Proceedings of the 13th international conference on Ubiquitous computing* (pp. 385-394). ACM.

75. Reid, S. C., Kauer, S. D., Hearps, S. J. C., Crooke, A. H. D., Khor, A. S., Sanci, L. A., & Patton, G. C. (2011). A mobile phone application for the assessment and management of youth mental health problems in primary care: a randomised controlled trial. *BMC Family Practice*, *12*(1), 131. https://doi.org/10.1186/1471-2296-12-131

76. Reid, S. C., Kauer, S. D., Hearps, S. J. C., Crooke, A. H. D., Khor, A. S., Sanci, L. A., & Patton, G. C. (2013). A mobile phone application for the assessment and management of youth mental health problems in primary care: health service

outcomes from a randomised controlled trial of mobiletype. *BMC Family Practice*, *14*, 84. https://doi.org/10.1186/1471-2296-14-84

77. Riva, G., Gorini, A., & Gaggioli, A. (2009). The Intrepid project - biosensor-enhanced virtual therapy for the treatment of generalized anxiety disorders. *Studies in Health Technology and Informatics*, *142*, 271–276.

78. Roesler, V., Binotto, A. P. D., Iochpe, C., Palomba, E. B., & Tizatto, L. A. (2015). Improving Preventive Healthcare with an User-centric Mobile Tele-monitoring Model. In *MedInfo* (pp. 648-652).

79. Roh, T., Song, K., Cho, H., Shin, D., & Yoo, H.-J. (2014). A wearable neuro-feedback system with EEG-based mental status monitoring and transcranial electrical stimulation. *IEEE Transactions on Biomedical Circuits and Systems*, *8*(6), 755–764. https://doi.org/10.1109/TBCAS.2014.2384017

80. Saeb, S., Zhang, M., Karr, C. J., Schueller, S. M., Corden, M. E., Kording, K. P., & Mohr, D. C. (2015). Mobile phone sensor correlates of depressive symptom severity in daily-life behavior: an exploratory study. *Journal of Medical Internet Research*, *17*(7), e175. https://doi.org/10.2196/jmir.4273

81. Schwenk, M., Hauer, K., Zieschang, T., Englert, S., Mohler, J., & Najafi, B. (2014). Sensor-derived physical activity parameters can predict future falls in people with dementia. *Gerontology*, *60*(6), 483–492. https://doi.org/10.1159/000363136

82. Soares Teles, A., Rocha, A., Jose da Silva E Silva, Francisco, Correia Lopes, J., O'Sullivan, D., van de Ven, P., & Endler, M. (2017). Enriching mental health mobile assessment and intervention with situation awareness. *Sensors (Basel, Switzerland)*, *17*(1). https://doi.org/10.3390/s17010127

83. Tighe, J., Shand, F., Ridani, R., Mackinnon, A., La Mata, N. de, & Christensen, H. (2017). Ibobbly mobile health intervention for suicide prevention in Australian Indigenous youth: a pilot randomised controlled trial. *BMJ Open*, *7*(1), e013518. https://doi.org/10.1136/bmjopen-2016-013518

84. Tsapeli, F., & Musolesi, M. (2015). Investigating causality in human behavior from smartphone sensor data: a quasi-experimental approach. *EPJ Data Science*, *4*(1), 337. https://doi.org/10.1140/epjds/s13688-015-0061-1

85. Wahle, F., Kowatsch, T., Fleisch, E., Rufer, M., & Weidt, S. (2016). Mobile sensing and support for people with depression: a pilot trial in the wild. *JMIR mHealth and uHealth*, *4*(3), e111. https://doi.org/10.2196/mhealth.5960

86. Wang, J. (2014). A wearable sensor (Fitbit One) and text-messaging to promote physical activity and participants' level of engagement (a randomized controlled feasibility trial). Dissertation, University of California, San Diego.

87. Whitehouse, S. R., Lam, P.-Y., Balka, E., McLellan, S., Deevska, M., Penn, D.,. . . Paone, M. (2013). Co-creation with TickiT: designing and evaluating a clinical eHealth platform for youth. *JMIR Research Protocols*, *2*(2), e42. https://doi.org/10.2196/resprot.2865

88. Whittaker, R., Merry, S., Stasiak, K., McDowell, H., Doherty, I., Shepherd, M.,. . . Rodgers, A. (2012). MEMO--a mobile phone depression prevention intervention for

adolescents: development process and postprogram findings on acceptability from a randomized controlled trial. *Journal of Medical Internet Research*, *14*(1), e13. https://doi.org/10.2196/jmir.1857

89. Whitton, A. E., Proudfoot, J., Clarke, J., Birch, M.-R., Parker, G., Manicavasagar, V., & Hadzi-Pavlovic, D. (2015). Breaking open the black box: isolating the most potent features of a web and mobile phone-based intervention for depression, anxiety, and stress. *JMIR Mental Health*, *2*(1), e3. https://doi.org/10.2196/mental.3573

90. Woo, Y. S., Bahk, W.-M., Hong, J., Yoon, B.-H., Hwang, T.-Y., Kim, M.-D., & Jon, D.-I. (2016). Use of a smartphone application to screen for bipolar spectrum disorder in a community sample. *Health Informatics Journal*, *22*(3), 779–788. https://doi.org/10.1177/1460458215589601

D) No monitoring of objective data involving a mobile device

1. Al-Asadi, A. M., Klein, B., & Meyer, D. (2014). Comorbidity structure of psychological disorders in the online e-PASS data as predictors of psychosocial adjustment measures: psychological distress, adequate social support, self-confidence, quality of life, and suicidal ideation. *Journal of Medical Internet Research*, *16*(10), e248. https://doi.org/10.2196/jmir.3591

2. Bauer, M., Wilson, T., Neuhaus, K., Sasse, J., Pfennig, A., Lewitzka, U.,. . . Whybrow, P. C. (2008). Self-reporting software for bipolar disorder: Validation of ChronoRecord by patients with mania. *Psychiatry Research*, *159*(3), 359–366. https://doi.org/10.1016/j.psychres.2007.04.013

3. Bauer, M., Grof, P., Gyulai, L., Rasgon, N., Glenn, T., & Whybrow, P. C. (2004). Using technology to improve longitudinal studies: self-reporting with ChronoRecord in bipolar disorder. *Bipolar Disorders*, *6*(1), 67–74.

4. Bopp, J. M. (2014). The longitudinal course of adolescent bipolar disorder as revealed through weekly self-report, using internet and text-messaging-based mood monitoring. *Psychology and Neuroscience Graduate Theses & Dissertations*, *68*.

5. Bopp, J. M., Miklowitz, D. J., Goodwin, G. M., Stevens, W., Rendell, J. M., & Geddes, J. R. (2010). The longitudinal course of bipolar disorder as revealed through weekly text messaging: a feasibility study. *Bipolar Disorders*, *12*(3), 327–334. https://doi.org/10.1111/j.1399-5618.2010.00807.x

6. Burton, C., Szentagotai Tatar, A., McKinstry, B., Matheson, C., Matu, S., Moldovan, R.,. . . Serrano Blanco, A. (2016). Pilot randomised controlled trial of Help4Mood, an embodied virtual agent-based system to support treatment of depression. *Journal of Telemedicine and Telecare*, *22*(6), 348–355.

7. Depp, C. A., Ceglowski, J., Wang, V. C., Yaghouti, F., Mausbach, B. T., Thompson, W. K., & Granholm, E. L. (2014). Augmenting psychoeducation with a mobile intervention for bipolar disorder: a randomized controlled trial. *Journal of Affective Disorders*, *174*, 23–30. https://doi.org/10.1016/j.jad.2014.10.053

8. Depp, C. A., Mausbach, B., Granholm, E., Cardenas, V., Ben-Zeev, D., Patterson, T. L.,. . . Jeste, D. V. (2010). Mobile interventions for severe mental illness: design and preliminary data from three approaches. *The Journal of Nervous and Mental Disease*, *198*(10), 175.

9. Depp, C. A., Moore, R. C., Dev, S. I., Mausbach, B. T., Eyler, L. T., & Granholm, E. L. (2016). The temporal course and clinical correlates of subjective impulsivity in bipolar disorder as revealed through ecological momentary assessment. *Journal of Affective Disorders*, *193*, 145–150. https://doi.org/10.1016/j.jad.2015.12.016

10. Ebert, D., Tarnowski, T., Gollwitzer, M., Sieland, B., & Berking, M. (2013). A transdiagnostic internet-based maintenance treatment enhances the stability of outcome after inpatient cognitive behavioral therapy: a randomized controlled trial. *Psychotherapy and Psychosomatics*, *82*(4), 246–256. https://doi.org/10.1159/000345967

11. Espinosa, H. D., Carrasco, A., Moessner, M., Caceres, C., Gloger, S., Rojas, G.,. . . Krause, M. (2016). Acceptability study of "Ascenso": an online program for monitoring and supporting patients with depression in Chile. *Telemedicine Journal and e-Health*, *22*(7), 577–583. https://doi.org/10.1089/tmj.2015.0124

12. Faurholt-Jepsen, M., Ritz, C., Frost, M., Mikkelsen, R. L., Margrethe Christensen, E., Bardram, J.,. . . Kessing, L. V. (2015). Mood instability in bipolar disorder type I versus type II-continuous daily electronic self-monitoring of illness activity using smartphones. *Journal of Affective Disorders*, *186*, 342–349. https://doi.org/10.1016/j.jad.2015.06.026

13. Fazzino, T. L., Rabinowitz, T., Althoff, R. R., & Helzer, J. E. (2013). Monitoring daily affective symptoms and memory function using interactive voice response in outpatients receiving electroconvulsive therapy. *The journal of ECT*, *29*(4), 318–324. https://doi.org/10.1097/YCT.0b013e3182972bbb

14. Forchuk, C., Donelle, L., Ethridge, P., & Warner, L. (2015). Client perceptions of the Mental Health Engagement Network: a secondary analysis of an intervention using smartphones and desktop devices for individuals experiencing mood or psychotic disorders in Canada. *JMIR Mental Health*, *2*(1), e1. https://doi.org/10.2196/mental.3926

15. Forchuk, C., Reiss, J. P., O'Regan, T., Ethridge, P., Donelle, L., & Rudnick, A. (2015). Client perceptions of the Mental Health Engagement Network: a qualitative analysis of an electronic personal health record. *BMC Psychiatry*, *15*(1), 250.

16. Forchuk, C., Reiss, J., Eichstedt, J., Singh, D., Collins, K., Rudnick, A.,. . . Fisman, S. (2016). The Youth-Mental Health Engagement Network: an exploratory pilot study of a smartphone and computer-based personal health record for youth experiencing depressive symptoms. *International Journal of Mental Health*, *45*(3), 205–222. https://doi.org/10.1080/00207411.2016.1204823

17. Guidi, A., Vanello, N., Bertschy, G., Gentili, C., Landini, L., & Scilingo, E. P. (2015). Automatic analysis of speech f0 contour for the characterization of mood changes in bipolar patients. *Biomedical Signal Processing and Control*, *17*, 29–37.

18. Hareva, D. H., Okada, H., Kitawaki, T., & Oka, H. (2009). Supportive intervention using a mobile phone in behavior modification. *Acta Medica Okayama*, *63*(2), 113–120.

19. Hidalgo-Mazzei, D., Mateu, A., Reinares, M., Murru, A., Del Mar Bonnin, C., Varo, C.,. . . Colom, F. (2016). Psychoeducation in bipolar disorder with a SIMPLe smartphone application: feasibility, acceptability and satisfaction. *Journal of Affective Disorders*, *200*, 58–66. https://doi.org/10.1016/j.jad.2016.04.042

20. Holmes, E. A., Bonsall, M. B., Hales, S. A., Mitchell, H., Renner, F., Blackwell, S. E.,. . . Di Simplicio, M. (2016). Applications of time-series analysis to mood fluctuations in bipolar disorder to promote treatment innovation: a case series. *Translational Psychiatry*, *6*(1), e720. https://doi.org/10.1038/tp.2015.207

21. Hung, S., Li, M.-S., Chen, Y.-L., Chiang, J.-H., Chen, Y.-Y., & Hung, G. C.-L. (2016). Smartphone-based ecological momentary assessment for Chinese patients with depression: an exploratory study in Taiwan. *Asian Journal of Psychiatry*, *23*, 131–136. https://doi.org/10.1016/j.ajp.2016.08.003

22. Joshi, J., Goecke, R., Alghowinem, S., Dhall, A., Wagner, M., Epps, J.,. . . Breakspear, M. (2013). Multimodal assistive technologies for depression diagnosis and monitoring. *Journal on Multimodal User Interfaces*, *7*(3), 217–228. https://doi.org/10.1007/s12193-013-0123-2

23. Kordy, H., Wolf, M., Aulich, K., Bürgy, M., Hegerl, U., Hüsing, J.,. . . Backenstrass, M. (2016). Internet-delivered disease management for recurrent depression: a multicenter randomized controlled trial. *Psychotherapy and Psychosomatics*, *85*(2), 91–98.

24. Migliorini, M. (2012). Study of heart rate variability in bipolar disorder: linear and nonlinear parameters during sleep. *Frontiers in Neuroengineering*, *4.* https://doi.org/10.3389/fneng.2011.00022

25. Miklowitz, D. J., Price, J., Holmes, E. A., Rendell, J., Bell, S., Budge, K.,. . . Geddes, J. R. (2012). Facilitated integrated mood management for adults with bipolar disorder. *Bipolar Disorders*, *14*(2), 185–197. https://doi.org/10.1111/j.1399-5618.2012.00998.x

26. Moore, P. J., Little, M. A., McSharry, P. E., Goodwin, G. M., & Geddes, J. R. (2014). Mood dynamics in bipolar disorder. *International Journal of Bipolar Disorders*, *2*(1), 1–9.

27. Palmier-Claus, J. E. (2011). 'Translating assessments of the film of daily life into person-tailored feedback interventions in depression': Reply. *Acta Psychiatrica Scandinavica*, *123*(5), 403–404. https://doi.org/10.1111/j.1600-0447.2011.01683.x

28. Pfeiffer, P. N., Valenstein, M., Ganoczy, D., Henry, J., Dobscha, S. K., & Piette, J. D. (2016). Pilot study of enhanced social support with automated telephone monitoring after psychiatric hospitalization for depression. *Social Psychiatry and Psychiatric Epidemiology*, 1–9. https://doi.org/10.1007/s00127-016-1288-2

29. Piette, J. D., Aikens, J. E., Trivedi, R., Parrish, D., Standiford, C., Marinec, N. S.,. . . Bernstein, S. J. (2013). Depression self-management assistance using automated

telephonic assessments and social support. *The American Journal of Managed Care*, *19*(11), 892–900.

30. Piette, J. D., Sussman, J. B., Pfeiffer, P. N., Silveira, M. J., Singh, S., & Lavieri, M. S. (2013). Maximizing the value of mobile health monitoring by avoiding redundant patient reports: prediction of depression-related symptoms and adherence problems in automated health assessment services. *Journal of Medical Internet Research*, *15*(7), e118. https://doi.org/10.2196/jmir.2582
31. Proudfoot, J., Parker, G., Hadzi Pavlovic, D., Manicavasagar, V., Adler, E., & Whitton, A. (2010). Community attitudes to the appropriation of mobile phones for monitoring and managing depression, anxiety, and stress. *Journal of Medical Internet Research*, *12*(5), e64. https://doi.org/10.2196/jmir.1475
32. Ramirez, M., Wu, S., Jin, H., Ell, K., Gross-Schulman, S., Myerchin Sklaroff, L., & Guterman, J. (2016). Automated remote monitoring of depression: acceptance among low-income patients in diabetes disease management. *JMIR Mental Health*, *3*(1), e6. https://doi.org/10.2196/mental.4823
33. Scherer, S., Stratou, G., Lucas, G., Mahmoud, M., Boberg, J., Gratch, J.,. . . Morency, L.-P. (2014). Automatic audiovisual behavior descriptors for psychological disorder analysis. *Image and Vision Computing*, *32*(10), 648–658. https://doi.org/10.1016/j.imavis.2014.06.001
34. Schwartz, S., Schultz, S., Reider, A., & Saunders, E. F. (2016). Daily mood monitoring of symptoms using smartphones in bipolar disorder: a pilot study assessing the feasibility of ecological momentary assessment. *Journal of Affective Disorders*, *191*, 88–93.
35. Sheeran, T., Rabinowitz, T., Lotterman, J., Reilly, C. F., Brown, S., Donehower, P.,. . . Bruce, M. L. (2011). Feasibility and impact of telemonitor-based depression care management for geriatric homecare patients. *Telemedicine and e-Health*, *17*(8), 620–626. https://doi.org/10.1089/tmj.2011.0011
36. Simons, C., Hartmann, J. A., Kramer, I., Menne-Lothmann, C., Höhn, P., van Bemmel, A. L.,. . . Wichers, M. (2015). Effects of momentary self-monitoring on empowerment in a randomized controlled trial in patients with depression. *European Psychiatry*, *30*(8), 900–906. https://doi.org/10.1016/j.eurpsy.2015.09.004
37. Sung, M., Marci, C., & Pentland, A. (2005). Objective physiological and behavioral measures for identifying and tracking depression state in clinically depressed patients. *Massachusetts Institute of Technology Media Laboratory, Cambridge, MA, Tech. Rep. TR*, *595*.
38. Thompson, W. K., Gershon, A., O'hara, R., Bernert, R. A., & Depp, C. A. (2014). The prediction of study-emergent suicidal ideation in bipolar disorder: a pilot study using ecological momentary assessment data. *Bipolar Disorders*, *16*(7), 669–677. https://doi.org/10.1093/oxfordhb/9780199381708.013.1
39. Torous, J., Staples, P., Shanahan, M., Lin, C., Peck, P., Keshavan, M., & Onnela, J.-P. (2015). Utilizing a personal smartphone custom app to assess the Patient Health Questionnaire-9 (PHQ-9) Depressive Symptoms in patients with major depressive disorder. *JMIR Mental Health*, *2*(1), e8. https://doi.org/10.2196/mental.3889

40. Trevino, A., Quatieri, T., & Malyska, N. (2011). Phonologically-based biomarkers for major depressive disorder. *EURASIP Journal on Advances in Signal Processing*, *2011*(1), 42. https://doi.org/10.1186/1687-6180-2011-42

41. Tsanas, A., Saunders, K. E. A., Bilderbeck, A. C., Palmius, N., Osipov, M., Clifford, G. D.,. . . Vos, M. de. (2016). Daily longitudinal self-monitoring of mood variability in bipolar disorder and borderline personality disorder. *Journal of Affective Disorders*, *205*, 225–233. https://doi.org/10.1016/j.jad.2016.06.065

42. Valenza, G., Gentili, C., Lanata, A., & Scilingo, E. P. (2013). Mood recognition in bipolar patients through the PSYCHE platform: preliminary evaluations and perspectives. *Artificial Intelligence in Medicine*, *57*(1), 49–58. https://doi.org/10.1016/j.artmed.2012.12.001

43. Valenza, G., Nardelli, M., Lanata, A., Gentili, C., Bertschy, G., Kosel, M., & Scilingo, E. P. (2016). Predicting mood changes in bipolar disorder through heartbeat nonlinear dynamics. *IEEE Journal of Biomedical and Health Informatics*, *20*(4), 1034–1043. https://doi.org/10.1109/JBHI.2016.2554546

44. Valenza, G., Nardelli, M., Lanata, A., Gentili, C., Bertschy, G., Paradiso, R., & Scilingo, E. P. (2014). Wearable monitoring for mood recognition in bipolar disorder based on history-dependent long-term heart rate variability analysis. *IEEE Journal of Biomedical and Health Informatics*, *18*(5), 1625–1635. https://doi.org/10.1109/JBHI.2013.2290382

45. Whybrow, P. C., Grof, P., Gyulai, L., Rasgon, N., Glenn, T., & Bauer, M. (2003). The electronic assessment of the longitudinal course of bipolar disorder: the ChronoRecord software. *Pharmacopsychiatry*, *36 Suppl 3*, S244-9. https://doi.org/10.1055/s-2003-45137

46. Wichers, M., Hartmann, J. A., Kramer, I. M. A., Lothmann, C., Peeters, F., van Bemmel, L., Myin-Germeys, I.,. . . Simons, C. J. P. (2011). Translating assessments of the film of daily life into person-tailored feedback interventions in depression. *Acta Psychiatrica Scandinavica*, *123*, 402–403. https://doi.org/10.1111/j.1600-0447.2011.01684.x

E) Other reasons

1. Andersen, P.-O. B., & Babic, A. (2013). Mobile-supported life charting for bipolar patients - user requirements study. *Studies in Health Technology and Informatics*, *192*, 1111.

2. Bahk, W., Hong, J. W., Woo, Y. S., & Yoon, B. H. (2012). P.2.e.007 Screening for bipolar disorder using a smartphone. *European Neuropsychopharmacology*, *22*, 279-S280. https://doi.org/10.1016/S0924-977X(12)70423-6

3. Bahk, W., Hong, J. W., Woo, Y. S., & Yoon, B. H. (2012). Screening for bipolar disorder using a smartphone. *Asia-Pacific Psychiatry*, *4*, 123. https://doi.org/10.1111/j.1758-5872.2012.00187.x

4. Ben-Zeev, D., Scherer, E. A., Wang, R., Xie, H., & Campbell, A. T. (2015). Next-generation psychiatric assessment: Using smartphone sensors to monitor behavior and mental health: Correction to Ben-Zeev et al. (2015). *Psychiatric Rehabilitation Journal*, *38*(4), 313.
5. Bitsch, J. A., Ramos, R., Ix, T., Ferrer-Cheng, P. G., & Wehrle, K. (2015). Psychologist in a pocket: towards depression screening on mobile phones. *Studies in Health Technology and Informatics*, *211*, 153–159.
6. Fernandez, K. C., Johnson, M. R., & Rodebaugh, T. L. (2013). Erratum to: TelEMA: a low-cost and user-friendly telephone assessment platform. *Behavior Research Methods*, *45*(4), 1292. https://doi.org/10.3758/s13428-013-0393-3
7. Frank, E., Matthews, M., Choudhury, T., Voida, S., & Abdullah, S. (2013, December). Developing a smart phone app to monitor mood, social rhythms, sleep and social activity: Technology to support effective management of bipolar disorder. In *NEUROPSYCHOPHARMACOLOGY* (Vol. 38, pp. S230-S231). MACMILLAN BUILDING, 4 CRINAN ST, LONDON N1 9XW, ENGLAND: NATURE PUBLISHING GROUP.
8. Gaggioli, A., & Riva, G. (2014). Psychological treatments: smart tools boost mental-health care. *Nature*, *512*(7512), 28. https://doi.org/10.1038/512028b
9. Hickie, I., Scott, E., & Merikangas, K. ([published online 2015]). Use of mobile technologies to monitor activity, sleep, and mood states to identify targets of prevention of mood disorders. *Neuropsychopharmacology.* Advance online publication. https://doi.org/10.1038/npp.2015.324
10. Hidalgo-Mazzei, D., Reinares, M., Murru, A., Bonnin, C. M., Vieta, E., & Colom, F. (2015). Signs and symptoms self-monitoring and psychoeducation in bipolar patients with a smart-phone application (SIMPLe) project. *European Psychiatry*, *30*, 320. https://doi.org/10.1016/S0924-9338(15)30255-8
11. Hidefjäll, P., & Titkova, D. (2015). Business model design for a wearable biofeedback system. Studies in Health Technology and Informatics, 211, 213–224.
12. Jacoby, A. S., Faurholt-Jepsen, M., Vinberg, M., Frost, M., Bardram, J., & Kessing, L. V. (2012). Electronic monitoring of patients with bipolar affective disorder [Elektronisk monitorering af patienter med bipolar affektiv sindslidelse]. *Ugeskrift for laeger*, *174*(44), 2707–2710.
13. Javelot, H., Spadazzi, A., Weiner, L., Garcia, S., Gentili, C., Kosel, M., & Bertschy, G. (2014). Telemonitoring with respect to mood disorders and information and communication technologies: overview and presentation of the PSYCHE project. *BioMed Research International*, *2014*, 104658. https://doi.org/10.1155/2014/104658
14. Ma, Y., Xu, B., Bai, Y., Sun, G., & Zhu, R. (2014). Infer daily mood using mobile phone sensing. *Ad Hoc & Sensor Wireless Networks*, *20*(1-2), 133–152.
15. Marcu, G., Bardram, J. E., Fauerholt-Jepsen, M., Vinberg, M., & Kessing, L. V. (2011). MONARCA: a mobile phone monitoring system to support episode detection and prevention in bipolar disorder patients. *Bipolar Disorders*, *13*(suppl. 1).

16. Mayora, O., Arnrich, B., Bardram, J., Dräger, C., Finke, A., Frost, M., ... & Haux, R. (2013, May). Personal health systems for bipolar disorder anecdotes, challenges and lessons learnt from monarca project. In *Pervasive computing technologies for healthcare (PervasiveHealth), 2013 7th international conference on* (pp. 424-429). IEEE.

17. Mertz, L. (2016). The end of seizures and depression?: New biosensors that warn of and potentially prevent health conditions. *IEEE Pulse*, *7*(1), 18–20. https://doi.org/10.1109/MPUL.2015.2498476

18. Naslund, J. A., Aschbrenner, K. A., Barre, L. K., & Bartels, S. J. (2015). Feasibility of popular m-Health technologies for activity tracking among individuals with serious mental illness. *Telemedicine and e-Health*, *21*(3), 213–216. https://doi.org/10.1089/tmj.2014.0105

19. Naveršnik, K., & Mrhar, A. (2014). Routine real-time cost-effectiveness monitoring of a web-based depression intervention: a risk-sharing proposal. *Journal of Medical Internet Research*, *16*(2), e67. https://doi.org/10.2196/jmir.2592

20. Valenza, G. & Scilingo E. P. (2014). Autonomic nervous system dynamics for mood and emotional-state recognition: Significant advances in data acquisition, signal processing and classification. Series in bioengineering. Cham, Switzerland: Springer International Publishing. ISBN: 978-3-319-02639-8.

21. Weidt, S., Wahle, F., Rufer, M., Hörni, A., & Kowatsch, T. (2015). MOSS: Mobile Sensing and Support Mit einer App depressive Verstimmungen erkennen und Betroffenen helfen. *Therapeutische Umschau*, *72*(9), 553–555. https://doi.org/10.1024/0040-5930/a000717

22. Whittaker, R., Merry, S., Dorey, E., & Maddison, R. (2012). A development and evaluation process for mHealth interventions: examples from New Zealand. *Journal of Health Communication*, *17 Suppl 1*, 11–21. https://doi.org/10.1080/10810730.2011.649103

23. Zhang, M. W., Ho, R. C., & Mcintyre, R. S. (2016). The `WikiGuidelines' smartphone application: Bridging the gaps in availability of evidence-based smartphone mental health applications. *Technology and Health Care*, *24*(4), 587–590. https://doi.org/10.3233/THC-161141
