# Supplementary material for: Smartphone-Based Monitoring of Objective and Subjective Data in Affective Disorders: Where Are We and Where Are We Going? Systematic Review
Source: J Med Internet Res. 2017 Jul 24;19(7):e262. doi: 10.2196/jmir.7006 (PMC5547249; doi:10.2196/jmir.7006)
Supplement: Multimedia Appendix 4 [file jmir_v19i7e262_app4.pdf]

Multimedia Appendix 4

Overview on selected studies

Reference<br><br>Country	Objective (as stated in the respective publication)	Used sensors and data obtained	Study design; number of participants; assessment duration	Results summary
**MONARCA** (Monitoring, treatment and prediction of bipolar disorder episodes)				
Bardram et al. (2013) [38]<br><br>Denmark	To establish the feasibility and usefulness of the MONARCA system	Subjective: Self-assessment items (e.g. mood, sleep duration, activity, medicine adherence)<br>Objective: Physical activity (accelerometer), social activity (in- & out-going calls & text messages)	Single arm observational design;<br>14 patients with BD (mean age 36 y);<br>14 weeks	Adherence rates for phone-based self-assessment were comparable with paper-based self-assessment. Higher compliance scores in phone-based self-assessments of mandatory items were observed. According to the usability scores measured by CSUQ overall usability of the system was good and the system was found very useful. The information quality score was lower. Interface quality was also scored well. The usefulness of the system for disease management during the trial scored 3.16 in average (on a scale from 1 to 7).
Frost et al. (2013) [43]<br><br>Denmark	To investigate if using data mining functionality in a system processing subjective and objective data from patients suffering from bipolar disorder would find relevant impact factors influencing moos and make sensible forecasting	Subjective: Self-monitored items (e.g. mood, sleep duration, activity, medicine adherence)<br>Objective: social activity (incoming and outgoing calls and text messages), physical activity (accelerometer), mobility (changes in cell tower IDs), phone usage (screen-on time, application usage)	Single arm observational design;<br>6 patients with BD (mean age not given);<br>6 weeks	An adherence rate of 91% for self-reporting was found. Self-assessed activity, stress and sleep as well as objectively measured phone usage had the highest rankings in the correlation between mood scores and impact factors. Including both objective and subjective data showed lower mean absolute error in 5 days forecast.

Alvarez-Lozano et al. (2014) [52]<br><br>Denmark (not clearly specified in the article)	To reveal factors correlating with smartphone usage; to understand the correlation between patient state and smartphone usage over time; and to investigate differen-ces in smartphone usage before and after psychiatric evaluation.	Subjective: Self-assessment items (e.g. mood, sleep duration, activity, medicine adherence)<br>Objective: smartphone usage (WiFi, running applications, screen status, Bluetooth connections, microphone)	Single arm observational design;<br>18 patients with BD (mean age not given);<br>5 months	Results showed strong correlations between app-usage patterns and self-reported states including mood, sleep and irritability. However, the app usage of patients changed discernibly in the period before and after psychiatric evaluation.
Faurholt-Jepsen et al. (2014) [26]<br><br>Denmark	Investigation of possible correlations between clinically rated depressive and manic symp-toms and subjective and objective smartphone data	Subjective: Self-assessment items (e.g. mood, sleep duration, activity, medicine adherence)<br>Objective: Speech duration, social activity (numbers of outgoing and incoming call and text messages/24 h), accelerometer, cell tower ID	Single arm observational design;<br>17 BD patients (mean age 33.4y);<br>3 months	Significant correlations between objective smartphone measures (physical and social activity) and clinically rated depressive symptoms were found. Self-monitored depressive symptoms correlated significantly and negatively with HDRS-17.
Faurholt-Jepsen et al. (2015) [39]<br><br>Denmark	To evaluate whether daily electronic self-monitoring reduces depressive and manic symptoms in patients with BD.	Subjective: (only in intervention group) Self-monitored items (e.g. mood, sleep duration, activity, medicine adherence).<br>Objective: Speech duration, social activity, physical activity and movement	Randomized single-blind placebo-controlled, parallel group trial;<br>78 patients with BD randomized, 67 analyzed (mean age 29.3y);<br>6 months	A tendency towards more sustained depressive symptoms in the intervention group (B = 2.02, 95% confidence interval -.13 to 4.17, *P* = .066) was found. During the trial period in the intervention group, significantly more depressive and fewer manic symptoms were shown in the sub-group analysis among patients without mixed symptoms and patients with presence of depressive and manic symptoms.

Faurholt-Jepsen et al. (2015) [41]<br><br>Denmark	To investigate if daily electronic self-monitored data and automatically generated objective data collected using smartphones correlate with clinical ratings of depressive and manic symptoms in patients with BD.	<u>Subjective</u>: (only in subgroup) Self-monitored items (e.g. mood, sleep duration, activity, medicine adherence)<br><u>Objective</u>: Social activity (e.g. number of incoming and outgoing calls/day; number of incoming and outgoing text messages/day)	Data taken from MONARCA I RCT [39]<br>61 BD patients (mean age 29.2y);<br>for 6 months	Via automatically generated objective data affective states could be differentiated: HDRS-17 scores showed significant positive correlations with duration of in- and outgoing calls/day and significant negative correlations with self-monitored data; YMRS scores showed positive correlations with number and duration of in- and outgoing calls/day as well as number of outgoing text messages/day. There were also significant positive correlations between self-monitored data YMRS scores.
Faurholt-Jepsen et al. (2016) [42]<br>Denmark	To investigate if automatically generated objective smartphone data correlates with the level of depressive and manic symp-toms and can be used to discriminate between categories of affective states in patients with BD presenting with more severe depressive and manic symptoms.	<u>Subjective</u>: Self-monitored items (e.g. mood, sleep duration, activity, medicine adherence)<br><u>Objective</u>: Social activity (e.g. number of incoming and outgoing calls/day; number of in- and outgoing text messages/day, number of missed calls); phone usage (duration and frequency of screen switched on); movement/ location (changes in cell tower IDs)	Single arm observational design;<br>29 patients with BD (mean age 30.2 y);<br>12 weeks	Automatically generated objective data on smartphone usage discriminated between mood states. 5 out of 13 variables correlated with HDRS-17 scores (patients with higher symptoms engaged less in communicative activities); 5 out of 13 variables correlated with YMRS scores (patients with more severe manic symptoms engaged more in communicative activities and moved around more). There were also significant positive correlations between self-monitored mood, acti-vity, stress levels and YMRS scores.

Faurholt-Jepsen et al. (2016) [40]<br><br>Denmark	To investigate voice features collected during phone calls as objective markers of affective states in bipolar disorder as well as if combining voice features with automatically generated objective smartphone data on behavioral activities and electronic self-monitored data on illness activity would increase the accuracy as a marker of affective states.	<u>Subjective</u>: Self-monitored items (e.g. mood, sleep duration, activity, medicine adherence)<br><u>Objective</u>: Acoustic voice features (extracted from patients' phone calls), social activity (e.g. number of incoming and outgoing calls/day; number of incoming and outgoing text messages/day, number of missed calls), phone usage (duration and frequency of screen switched on), mobility/location (changes in cell tower IDs)	Single arm observational design;<br>28 patients with BD (mean age 30.3 y);<br>12 weeks	Mood states of bipolar patients can be classified by models based exclusively on voice features extracted during real-life phone calls in naturalistic settings.<br>Manic or mixed states were classified more accurate, specific and sensitive by voice features as compared with depressive states. Combination of voice features, self-monitored data and objective data increased the accuracy, specificity and sensitivity in classification models of mood sates.
Maxhuni et al. (2016) [53]<br><br>Austria	To evaluate the performance of different sets of features in classifying bipolar disorder episodes based on audio, accelerometer and self-assessment data	<u>Subjective</u>: Daily self-assessment<br><u>Objective</u>: acoustic voice features, accelerometer, magnetometer, GPS, Wi-Fi Access Points, Bluetooth, phone usage, social activity (number and duration of phone calls, number and length of SMS)	Single arm observational design;<br>10 patients with BD, data of only 5 patients analyzed (mean age not given);<br>12 weeks	The information obtained from the frequency domain features of the accelerometers lead to higher classification accuracy than the information extracted from audio. The frequency domain features also produced better classification results than the time domain features of the accelerometers.<br>Personalized models behave better than single models that combined information from all patients.
**Applications associated with MONARCA-project**				
Grünerbl et al. (2012) [54]<br>Austria	To investigate if sufficient information can be extracted from sensors contained in a smartphone to reliably support diagnosis and prediction of episode changes.	<u>Subjective</u>: Daily self-assessment.<br><u>Objective</u>: GPS, accelerometer, social interaction	Single arm observational design;<br>10 patients with BD (age range 33-48y);<br>for 6 to 8 weeks	Simple features obtained from location, physical activity and phone call patterns showed to be good indicators for mood state classification.

Osmani et al. (2013) [55]<br><br>Austria	To investigate associations between physical activity levels measured through a smartphone with bipolar disorders episodes	Subjective: questionnaires on current state, sleep, social activity (not used in study)<br>Objective: Microphone, accelerometer, GPS, WiFi access points, Bluetooth, calls, SMS and their duration	Single arm observational design;<br>9 patients with BD (mean age not given);<br>for 3 months	(Paper reports only about physical activity): Correlation between physical activity levels during daily intervals and psychiatric assessment scores was much higher compared with the correlation of overall physical activity level and psychiatric assessment score.
Grünerbl et al. (2014) [56]<br><br>Austria	To demonstrate how smartphone usage patterns and sensor data can be used for aiding psychiatric care.	Subjective: none (clinicians ratings outside the application)<br>Objective: Phone features, speech features, voice features, GPS data, acceleration	Single arm observational design;<br>10 patients with BD (mean age not given);<br>for 12 weeks	Recognition accuracies of 76% and over 97% of state change detection precision and recall were gained by fusing all sensor modalities.
Grünerbl et al. (2014) [57]<br><br>Austria	To demonstrate how smartphone sensors can be used for aiding psychiatric diagnosis.	Subjective: Self-assessment questionnaires.<br>Objective: GPS, accelerometer	Single arm observational design;<br>12 patients with BD (mean age not given);<br>for 12 weeks (or more)	State recognition accuracy of 80% was found. Additionally, state change detection with a precision/recall of 96%/94% was achieved.
Muaremi et al. (2014) [58]<br><br>Austria	To explore the potential of smartphones for monitoring bipolar patients by focusing on voice analysis	Subjective: Daily self-assessment<br>Objective: phone call statistics (number, duration, time of day), social signal processing (speaking length, number of speaker turns, short utterances, etc.), acoustic voice features (from phone calls)	Single arm observational design;<br>12 patients with BD (mean age not given);<br>12 weeks or more	Mood states were correctly detected using a combination of all features with an average accuracy of 83 %. From the three categories, acoustic features showed best performance in terms of state recognition followed by the social cues. The speaking length and phone call length, the HNR value, the number of short turns/utterances and the pitch F0 were identified to be the most important variables.
**PSYCHE** (Personalized monitoring system for care in mental health)				
Lanata et al. (2015) [45]<br><br>Italy	To validate the PSYCHE system in terms of supporting the diagnosis and helping in the prognosis of patients affected by severe BD.	Subjective: mood, sleep, and other questionnaires<br>Objective: sensorized t-shirt recording ECG/HRV, respiration activity and movement activity (accelerometer); plus speech features	Single arm observational design;<br>10 patients with BD (mean age not given);<br>up to 6 recordings (max. 18h) per patient	Using only HRV parameters, it was shown that patients experiencing mood state changes from a pathological mood state to euthymia can be characterized through a measure of entropy.

Guidi et al. (2015) [46]<br><br>Italy & France	To explore the possibility of using a smartphone to collect and process speech data for the estimation of features related to the speech F0 and its variability; to present a correlatio-nal analysis among speech features and observed mood changes	Subjective: mood, sleep, and other questionnaires (not used in analysis though)<br>Objective: acoustic voice features (from 2 structured tasks)	Observational case report ;<br>1 patient with BD (36 y);<br>14 weeks	The quality of audio acquisitions from smartphone devices can be used to estimate different features describing the speech F0. The mean value of F0 estimated for each voice segment can be reliably obtained.<br>In the case study the variability of average F0 increased passing from hypomania to depression, yet no correlations were found among the estimated voice features and QIDS-C or YMRS scales.
Gentili et al. (2017) [47]<br><br>Italy & France	To determine whether alterations in the HRV of BD patients are a mere expression of the current mood state or rather contain longitudinal information on BD course,	Subjective: none (mood states rated by clinicians)<br>Objective: sensorized t-shirt recording ECG/HRV, respiration activity and movement activity (accelerometer)	Single arm observational design;<br>8 patients with BD (mean age 40 y);<br>between 3 and 6 recordings (min 300 h) per patient	Normalization of HRV series using information from future and previous mood states provided a significantly higher average classification accuracy (mean value 99.52+/-1) in automatic labeling of the patient mood states as compared to the independent characterization and other normalization procedures.
**SIMBA** (Social Information Monitoring for Patients with Bipolar Affective Disorders)				
Beiwinkel et al. (2016) [48]<br><br>Germany	To investigate whether smartphone measurements predicted clinical symptoms levels and clinical symptom change.	Subjective: Self-reported mood states.<br>Objective: GPS, accelerometer, cell tower movement, screen state, social communication (the number and duration of ongoing calls and the number of SMS sent per day)	Single arm observational design;<br>14 patients with BD (mean age 47.2y);<br>up to 12 months	Relationship between smartphone measures and overall symptom levels and change in clinical symptoms were found. Lower self-reported mood was a predictor for higher levels of clinical depressive symptoms. As increase in clinical depressive symptoms was associated with a decline in social communication and a decline in physical activity, an increase in clinical manic symptoms was related to a decrease in physical activity. Lower physical activity and higher social communication were predictive for higher levels of clinical manic symptoms were predicted by.

**Mobilyze!**				
Burns et al. (2011) [37]<br><br>USA	To investigate the technical feasibility, functional reliability, and patient satisfaction of a mobile phone- and Internet-based intervention including ecological momentary intervention and context sensing	Subjective: Self-reported mood states<br>Objective: GPS, accelerometer, ambient light, Wi-Fi, Bluetooth detection of other wireless devices, information from phone`s operating system (recent calls, apps)	Single arm observational design;<br>8 adults with MDD (mean age 37.4y);<br>over 8 weeks	Accuracy rates between 60% and 80% were achieved for predicting categorical states (e.g. location) but predictive capability was poor for states rated on scales (e.g. mood). Depressive symptoms (PHQ-9, QIDS-C) and MDD diagnostic status (MINI) decreased.
**MoodRhythm**				
Abdullah et al. (2016) [44]<br><br>USA	To overcome the limitations of existing self-reporting methods by developing a smartphone-based application for helping patients with BD maintaining stability and rhythmicity.	Subjective: ratings via SRM-5 and scales for mood and energy.<br>Objective: Light sensor, accelerometers, microphone, communication patterns (including SMS and call logs), phone usage, GPS, Wi-Fi, cellular data.	Single arm observational design;<br>7 patients with BD (age range 25-64y)<br>4 weeks	The generalized model (using location, distance travelled, conversation frequency, and non-stationary duration as inputs) achieved a reasonable performance with SRM-5 score as outcome measure (SRM-5 score (0-7): Prediction of stable (>3.5) and unstable (<3.5) states with high accuracy (precision: 0.85 and recall: 0.86).
**Empath** (Emotional Monitoring for Pathology)				
Dickerson et al. (2011) [34]<br><br>USA	To develop a real-time depression monitoring system for the home collecting multi-modal data; to show an example of the system in operation and how it is able to collect data	Subjective: scale for mood via smartphone, scores from the items in the CES-D via touchpad<br>Objective: sleep (mattress with accelerometer sensor motes), weight, acoustic voice features (prompted free speech response), activity (motion sensors), activities of daily living (sensors attached to appliances and hygiene facilities)	Observational case report ;<br>1 patient with MDD (age not given);<br>2 weeks	The Empath system could be deployed in a real apartment and collect data over the course of 2 weeks; there was a correlation between subjective sleep quality and measurement-based sleep-quality index; there also was a correlation between self-reported mood and a linear model of speech sample parameters; no relationship between mood and apartment occupancy could be shown

		**FINE**		
Dang et al. (2016) [35]<br><br>Germany	To develop and evaluate a concept for smartphone-based depression support collecting numerous types of data that may reveal behavioral factors	Subjective: self-assessment (PHQ-9) and mood diary<br>Objective: phone usage (e.g. screen status), communication (e.g. incoming and outgoing calls), movement (accelerometer, GPS location)	Single arm observational design;<br>4 patients with MDD (age range 27-46);<br>1 week	Recording most of the selected parameters via the app was found to be reliable. The overall concept has been accepted well, Further improvements are planned.
		**MedLink**		
Mohr et al. (2015) [36]<br><br>USA	To investigate the functional reliability and patient satisfaction with a mobile intervention consisting of an app and an electronic pill bottle; to evaluate adherence and depression severity in users of the intervention	Subjective: self-assessments of depressive symptoms (PHQ-8) and side effects (FIBSER)<br>Objective: medication adherence (using an electronic pill bottle	Single arm observational design;<br>8 patients with MDD (mean age 39.6 y);<br>4 weeks	Patients were moderately depressed at baseline, (PHQ-9=12.5), dropping after 4 weeks to mild (PHQ-9). Patients completed 96.6% of the in-app depression assessments and their results were nearly identical to the PHQ results obtained for research outcomes. Consistency of adherence, monitored using the pillbox, indicated that a mean 84% of doses were taken. There were issues with the reminder function due to connectivity problems with the cellularly enabled pill bottle.
		**others**		
Prociow et al. (2012) [59]<br><br>England	To evaluate the performance of a sensor network as well as to assess its potential for monitoring behavioral patterns in BD patients	Subjective: none<br>Objective: physical activity (accelerometer), location (GPS, Bluetooth), social activity (Bluetooth encounters), ambient light, acoustic voice features (microphone), sleep and activity monitoring via environmental sensors	Observational case report;<br>4 healthy subjects, 1 patient with BD (age not given);<br>Duration: not stated	The wearable and environmental sensors provided useful data, Behavioral data gathered through these sensors used in the study may be of significant use in detecting early signs and changes of a bipolar episode. Adherence was found lower in the patient trial.

Kane et al (2013) [50]<br><br>USA	To characterize the feasibility and safety of a wireless networked system incorporating physiologic assessments and direct confirmation of digital tablet ingestions in ambulatory patients with schizophrenia or bipolar disorder.	<u>Subjective</u>: Self-assessed sleep quality<br><u>Objective</u>: ingestion and physiological metrics (heart rate, body position, physical activity and sleep characteristics) via an ingestion sensor	Single arm observational design;<br>28 patients (12 BD, 16 schizo-phrenia) (mean age 42.8 y);<br>4 weeks	The mean adherence rate was 74%, as 67% timing adherence rate for doses that were taken within 2 hours of the prescribed dosing time was reported. Physiologic metrics (e.g., sleep duration) sleep duration were examined and quantified. Minor skin irritation was reported by five subjects which was the most common adverse effect documented. 70% of participants found the concept of the system easy to understand, while 89% reported that the system could be useful to them and 78% said that they would prefer to receive reminders.
Karam et al. (2014) [60]<br><br>USA	To test the hypothesis that speech collected in a setting outside of clinician interaction can be used to assess the underlying mood state.	<u>Subjective</u>: none (mood states rated by clinicians using HAMD and YMRS)<br><u>Objective</u>: acoustic voice features (extracted from the outgoing speech of patients' phone calls, as well as patients' speech in clinical interviews)	Single arm observational design;<br>6 patients with BD (mean age 41 y);<br>6 months to one year	Hypomania and depression can be differentiated from euthymia using speech-based classifiers trained on both structured (the weekly clinical interactions) and unstructured (all other calls) cell phone recordings. The accuracy is higher in calls recorded during the clinical interactions with an AUC of 0.81 for hypomania and 0.67 for depression
Gideon et al. (2016) [61]<br><br>USA	To investigate methodologies for use during pre-processing, feature extraction, and data modeling of speech audio recordings on two different phones with different sound quality for analysis of mood in patients with BD.	<u>Subjective</u>: none (mood states rated by clinicians using HAMD and YMRS)<br><u>Objective</u>: acoustic voice features (extracted from patients' phone calls)	Quasi-experimental design;<br>37 patients with rapid cycling BD (mean age not given);<br>6-12 months	It is possible to differentiate between euthymic and symptomatic moods, despite using two types of mobile phones with different acoustics. Preprocessing the audio data can improve the test performance from the baseline AUCs of 0.57 ± 0.25 for manic and 0.64 ± 0.14 for depressed to the significantly higher AUCs of 0.72 ± 0.20 and 0.75 ± 0.14, respectively.

Naslund et al. (2016) [51]<br><br>USA	To assess the acceptability of using wearable devices and smartphones to support a lifestyle intervention targeting weight loss in participants with serious mental illness.	Subjective: none (quantitative usability and satisfaction questionnaire and qualitative interview at the end of the intervention)<br>Objective: physical activity (accelerometer)	Single arm observational design;<br>11 patients with obesity and serious mental illness (3 schizophre-nia, 5 MDD, 3 BD) (mean age 48.2 y);<br>6 months	Encouragement for being more physically active as well as high satisfaction and usability were reported by participants. Using smartphone and companion mobile app nearby Fitbit was listed as a challenge by participants.
Saunders et al. (2017) [49]<br><br>England	To explore the experiences of individuals with bipolar disorder concerning mood and activity monitoring with a range of portable and wearable technologies.	Subjective: within in the AMoSS study daily (Mood Zoom) and weekly (True Colours) mood measurements;<br>Objective: physical activity (accelerometer), other physiological variables	Single arm observational design;<br>21 patients with BD (mean age 44.4 y) partaking in the AMoSS study; 12 weeks	Mood monitoring and actigraphy was well accepted and tolerated by individuals with BD. Monitoring was related with increased illness insight and behavioral change. Concerns were raised about the misinterpretation of data as illness relapse. Compliance with both questionnaires (Mood Zoom & True Colours) was high. The study devices were well tolerated by the majority of participants.

Abbreviations: AUC = area under the curve; BD = Bipolar Disorder; CSUQ = Computer System Usability Questionnaire; HDRS = Hamilton Depression Rating Scale; YMRS = Young Mania Rating Scale; RCT = randomized controlled trial; GPS = global positioning system; SMS = short message service; ECG = electrocardiogram; HRV = heart rate variability; MDD = Major Depressive Disorder; PHQ = Patient Health Questionnaire; QIDS-C = Quick Inventory of Depressive Symptomatology (clinician rated); MINI = Mini-International Neuropsychiatric Interview; SRM-5 = Social Mood Rhythm Metric
