# Supplementary material for: Smartphone-Based Monitoring of Objective and Subjective Data in Affective Disorders: Where Are We and Where Are We Going? Systematic Review
Source: J Med Internet Res. 2017 Jul 24;19(7):e262. doi: 10.2196/jmir.7006 (PMC5547249; doi:10.2196/jmir.7006)
Supplement: Multimedia Appendix 5 [file jmir_v19i7e262_app5.pdf]

Multimedia Appendix 4

Checklist for measuring study quality according to Downs and Black (1998):

From: Downs SH & Black N (1998). The feasibility of creating a checklist for the assessment of the methodological quality both of randomised and non-randomised studies of health care interventions. J Epidemiol Community Health 1998;52:377–384.

1. Is the hypothesis/aim/objective of the study clearly described? *[yes = 1, no = 0]*
2. Are the main outcomes to be measured clearly described in the Introduction or Methods section? *[yes = 1, no = 0]*
3. Are the characteristics of the patients included in the study clearly described? *[yes = 1, no = 0]*
4. Are the interventions of interest clearly described? *[yes = 1, no = 0]*
5. Are the distributions of principal confounders in each group of subjects to be compared clearly described? *[yes = 2, partly = 1, no = 0]*
6. Are the main findings of the study clearly described? *[yes = 1, no = 0]*
7. Does the study provide estimates of the random variability in the data for the main outcomes? *[yes = 1, no = 0]*
8. Have all important adverse events that may be a consequence of the intervention been reported? *[yes = 1, no = 0]*
9. Have the characteristics of patients lost to follow-up been described? *[yes = 1, no = 0]*
10. Have actual probability values been reported (e.g. 0.035 rather than <0.05) for the main outcomes except where the probability value is less than 0.001? *[yes = 1, no = 0]*
11. Were the subjects asked to participate in the study representative of the entire population from which they were recruited? *[yes = 1, no = 0, unable to determine = 0]*
12. Were those subjects who were prepared to participate representative of the entire population from which they were recruited? *[yes = 1, no = 0, unable to determine = 0]*
13. Were the staff, places, and facilities where the patients were treated, representative of the treatment the majority of patients receive? *[yes = 1, no = 0, unable to determine = 0]*
14. Was an attempt made to blind study subjects to the intervention they have received? *[yes = 1, no = 0, unable to determine = 0]*
15. Was an attempt made to blind those measuring the main outcomes of the intervention? *[yes = 1, no = 0, unable to determine = 0]*
16. If any of the results of the study were based on "data dredging", was this made clear? *[yes = 1, no = 0, unable to determine = 0]*
17. In trials and cohort studies, do the analyses adjust for different lengths of follow-up of patients, or in case-control studies, is the time period between the intervention and outcome the same for cases and controls? *[yes = 1, no = 0, unable to determine = 0]*

18. Were the statistical tests used to assess the main outcomes appropriate? *[yes = 1, no = 0, unable to determine = 0]*
19. Was compliance with the intervention/s reliable? *[yes = 1, no = 0, unable to determine = 0]*
20. Were the main outcome measures used accurate (valid and reliable)? *[yes = 1, no = 0, unable to determine = 0]*
21. Were the patients in different intervention groups (trials and cohort studies) or were the cases and controls (case-control studies) recruited from the same population? *[yes = 1, no = 0, unable to determine = 0]*
22. Were study subjects in different intervention groups (trials and cohort studies) or were the cases and controls (case-control studies) recruited over the same period of time? *[yes = 1, no = 0, unable to determine = 0]*
23. Were study subjects randomised to intervention groups? *[yes = 1, no = 0, unable to determine = 0]*
24. Was the randomised intervention assignment concealed from both patients and health care staff until recruitment was complete and irrevocable? *[yes = 1, no = 0, unable to determine = 0]*
25. Was there adequate adjustment for confounding in the analyses from which the main findings were drawn? *[yes = 1, no = 0, unable to determine = 0]*
26. Were losses of patients to follow-up taken into account? *[yes = 1, no = 0, unable to determine = 0]*
27. Did the study have sufficient power to detect a clinically important effect where the probability value for a difference being due to chance is less than 5%? *[yes = 1, no = 0] (NB: scoring of this item had been adapted)*

Methodological quality scores using Downs and Black Instrument

Study	1	2	3	4	5	6	7	8	9	10	11	12	13	14	15	16	17	18	19	20	21	22	23	24	25	26	27
Abdullah et al., 2016 [44]	1	1	1	1[a]	1	1	1	0[a]	1	0	0	0	1	0[a]	0[a]	1	1	1	0[a]	1[b]	1[b]	0[b]	0[b]	0[b]	0	1	0
Alvarez-Lozano et al., 2014 [52]	1	1[c]	0	1[a]	0	0	0[d]	0[a]	1	0[d]	0	0	0	0[a]	0[a]	1	0	1[d]	0[a]	1	0[b]	1[b]	0[b]	0[b]	0	0[e]	0
Bardram et al., 2013 [38]	1	1[c]	1	1[a]	1	1	1[d]	0[a]	1	0[d]	0	0	0	0[a]	0[a]	1	1	1	1	1	1[b]	1[b]	0[b]	0[b]	0	0[e]	0
Beiwinkel et al., 2016 [48]	1	1	1	1[a]	2	1	1	0[a]	1	1	0	0	1	0[a]	0[a]	1	1	1	1	1	1[b]	1[b]	0[b]	0[b]	1	1	0
Burns et al., 2011 [37]	1	1	1	1	2	1	1	0	1	1	0	0	0	0	0	1	1	1	1	1	1[b]	0[b]	0[b]	0[b]	0	1	0
Dang et al., 2016 [35]	1	1	0	1[a]	0	0	0[d]	0[a]	0	0[d]	0	0	1	0[a]	0[a]	0	1	0[d]	0[a]	1	1[b]	0[b]	0[b]	0[b]	0	0	0
Dickerson et al., 2011 [34]	1	1	0[f]	1[a]	0	1	1	0[a]	1	1	0	0	1	0[f]	0[f]	1	1[f]	1[f]	1[f]	1	0[f]	0[f]	0[f]	0[f]	0	1	0
Faurholt-Jepsen et al., 2014 [26]	1	1	1	1[a]	2	1	1	0[a]	1	1	0	0	0	0[a]	1[a]	1	1	1	1[a]	1	1[b]	1[b]	0[b]	0[b]	1	1	0
Faurholt-Jepsen et al., 2015 [39]	1	1	1	1	2	1	1	0	1	1	0	0	0	0	1	1	1	1	1	1	1	1	1	0	1	1	1
Faurholt-Jepsen et al., 2015 [41]	1	1	1	1	2	1	1	0	1	1	0	0	0	0	1	1	1	1	0	1	1	1	1	0	1	1	1
Faurholt-Jepsen et al., 2016 [42]	1	1	1	1[a]	2	1	1	0[a]	1	1	0	1	0	0[a]	1[a]	1	1	1	0	1	1[a]	1[a]	0[b]	0[b]	1	1	0
Faurholt-Jepsen et al., 2016 [40]	1	1	1	1[a]	2	1	1	0	1	0	0	0	0	0	1[a]	1	1	1	0	1	1[a]	1[a]	0	0	0	1	0
Frost et al., 2013 [43]	0	0[c]	0	1[a]	0	0	0	0[a]	0	0[d]	0	0	0	0[a]	0[a]	1	1	0	1	0	0[b]	1[b]	0[b]	0[b]	0	0	0
Gentili et al., 2017 [47]	1	1	1	1[a]	2	1	1	0[a]	0	1	0	0	1	0[a]	0[a]	1	0	1[d]	0	1	0[b]	0[b]	0[b]	0[b]	0	0[e]	0
Gideon et al., 2016 [61]	0	1[c]	1	1[a]	0	1	1	0[a]	1	0	0	0	1	0[a]	0[a]	1	0	1	0	1	1[b]	0[b]	0[b]	0[b]	0	0	0
Grünerbl et al., 2014 [57]	1	0[c]	1	1[a]	0	0	1[d]	0[a]	0	0[d]	0	0	1	0[a]	0[a]	1	0	1[d]	0[a]	1	1[b]	0[b]	0[b]	0[b]	0	0[e]	0

Study	1	2	3	4	5	6	7	8	9	10	11	12	13	14	15	16	17	18	19	20	21	22	23	24	25	26	27
Grünerbl et al., 2012 [54]	1	1[c]	0	1[a]	1	0	0[d]	0	1	0[d]	0	0	0	0[a]	0[a]	1	0	1	0	1	0[b]	0[b]	0[b]	0[b]	0	0	0
Gruenerbl et al., 2014 [56]	1	1[c]	1	1[a]	1	1	1[d]	0[a]	1	0[d]	0	0	1	0[a]	0[a]	1	0	1[d]	0[a]	1	1[b]	1[b]	0[b]	0[b]	0	0[e]	0
Guidi et al., 2015 [46]	1	1	1[f]	1[a]	0	1	1	0[a]	1	0[d]	0	0	1	0[f]	0[f]	1	1[f]	1[f]	0[f]	1	1[f]	0[f]	0[f]	0[f]	0	0[f]	0[f]
Kane et al., 2013 [50]	1	1	1	1[a]	2	1	1	1[a]	1	0	0	0	1	0[a]	0[a]	1	1	1	0	1	1[b]	0[b]	0[b]	0[b]	0	1	0
Karam et al., 2014 [60]	1	1	1	1[a]	1	1	1	0[a]	0	0	0	0	1	0[a]	0[a]	1	0	1	0	1	1[b]	1[b]	0[b]	0[b]	0	0[e]	0
Lanata et al.,2015 [45]	1	0	1	1[a]	0	1	1[d]	0[a]	0	0[d]	0	0	1	0[a]	0[a]	1	0	1[d]	0	0	1[b]	0[b]	0[b]	0[b]	0	0	0
Maxhuni et al., 2016 [53]	0	1[c]	1	1[a]	0	1	1[d]	0[a]	0	0[d]	0	0	1	0[a]	0[a]	1	0	1[d]	0[a]	1	1[b]	0[b]	0[b]	0[b]	0	0[e]	0
Mohr et al., 2015 [36]	1	1	1	1	1	1	1	0	1	1	0	0	1	0	0	1	0	1	1	1	1[b]	0[b]	0[b]	0[b]	0	1	0
Muaremi et al., [58]	1	0[c]	0	1[a]	0	1	1[d]	0[a]	1	0[d]	0	0	1	0[a]	0[a]	1	0	1[d]	0[a]	0	1[b]	0[b]	0[b]	0[b]	0	0[e]	0
Naslund et al., 2016 [51]	1	1	1	1[a]	2	1	1	0[a]	1	0[d]	0	0	1	0[a]	0[a]	1	1	0	1[a]	1	0	0[b]	0[b]	0[b]	0	1	0
Osmani et al., 2013 [55]	0	1[c]	1	1[a]	0	1	0[d]	0[a]	0	0[d]	0	0	1	0[a]	0[a]	1	1	1[d]	0[a]	1	0[b]	0[b]	0[b]	0[b]	0	0[e]	0
Prociow et al., 2012 [59]	1	1	0[f]	1[a]	0	0	0	0[a]	1	0[d]	0	0	0	0[f]	0[f]	1	1	0	0	0	0[f]	0[f]	0[f]	0[f]	0	1	0
Saunders et al., 2017 [49]	1	1	0	1[a]	2	1	1	0[a]	1	1	0	0	1	0[a]	0[a]	1	0	1[d]	1[a]	1	1[b]	0[b]	0[b]	0[b]	0	0	0

[a] - no clinical intervention, scoring of items asking about an intervention was performed with regard to the use of the respective self-monitoring tool; [b] - Only one group was investigated; [c] - Publication is not organized in the classical sections (introduction, methods, results, discussion); [d] - Results of individual patients are reported / no inferential statistics were done; [e] - Only compliant subjects with sufficient data were included in analyses; [f] - Case report
